# Discovery of Novel Iminosugar Compounds Produced by *Lactobacillus paragasseri* MJM60645 and Their Anti-Biofilm Activity against Streptococcus mutans

**DOI:** 10.1128/spectrum.01122-22

**Published:** 2022-07-06

**Authors:** Mingkun Gu, Jinhua Cheng, Yeong-Geun Lee, Joo-Hyung Cho, Joo-Won Suh

**Affiliations:** a Interdisciplinary Program of Biomodulation, Myongji University, Yongin, Republic of Korea; b Myongji Bioefficacy Research Center, Myongji University, Yongin, Republic of Korea; c Department of Oriental Medicine Biotechnology, College of Life Sciences, Kyung Hee University, Yongin, Republic of Korea; University of Florida

**Keywords:** *Lactobacillus paragasseri*, iminosugar, *Streptococcus mutans*, biofilm, oral microbiota

## Abstract

The oral cavity contains a number of microbes. They interact with each other and play an important role in human health. Among oral cariogenic microbes, Streptococcus mutans is recognized a major etiological bacteria of dental caries. Lactobacilli strains have been promoted as possible probiotic agents against S. mutans. However, their inhibitory mechanism has not been well elucidated yet. In the present study, two new compounds with strong antibiofilm activities were purified from the culture supernatant of Lactobacillus paragasseri MJM60645, which was isolated from the human oral cavity. These compounds showed strong inhibitory activities against S. mutans biofilm formation, with IC_50_ (concentration at which 50% biofilm was inhibited) of 30.4 μM for compound 1 and 18.9 μM for compound 2. However, these compounds did not show bactericidal activities against S. mutans. Structure elucidation by nuclear magnetic resonance (NMR) and mass spectrometry showed that compound 1 was composed of two arabinofuranose iminosugars jointed with one glycerol and oleic acid, and compound 2 was composed of two arabinofuranose iminosugars jointed with one glycerol and nervonic acid. To the best of our knowledge, these structures were discovered for the first time in this study. Treatment of S. mutans with compound 1 strongly downregulated expression levels of genes related to biofilm formation, including *gtfB*, *gtfC*, *gtfD*, *gbpB*, *brpA*, *spaP*, *ftf*, and *smu0630* without affecting the expression of *comDE* or *relA*. This study provides new insights into novel molecules produced by Lactobacillus to regulate the pathogenesis of S. mutans, facilitating a better understanding of the mechanism for interactions between Lactobacillus and S. mutans.

**IMPORTANCE** In this study, we isolated lactic acid bacteria that inhibit streptococcal biofilm from the oral cavity of infants and identified two novel compounds from the supernatant of their culture broth. The two compounds are structurally similar, and both consist of iminosugars, glycerol, and unsaturated fatty acid. A search of the SciFinder database revealed that these structures are novel and were discovered for the first time in this study. Mechanism studies have shown that these compounds can inhibit the expression of biofilm synthesis-related genes. This is the first report that lactic acid bacteria inhibit streptococcal biofilms by small molecules with new chemical structures. This study not only expands the understanding of natural products derived from lactic acid bacteria but also provides a new paradigm for the understanding of the interaction of bacteria in the oral microbiota.

## INTRODUCTION

Dental caries is one of the most common infectious diseases in humans and is closely related to human cardiovascular disease (1). It is a result of the homeostatic imbalance of oral microbiota. Although different bacteria are associated with the pathogenesis of dental caries, the mutans streptococcal group represented by Streptococcus mutans is considered to be a major cariogenic bacteria in oral cavity (2). S. mutans can colonize the hard tissues of tooth surface and mediate cariogenic biofilm formation, which enables them to survive in harsh environments.

Biofilm is a complex structure of bacteria consortium and extracellular polymeric matrix, consisting mainly of polysaccharides, proteins, and nucleic acids produced by bacteria or fungi (3). The so-called pathogenic biofilm is considered one of the most important and difficult to cure forms of microbial pathogenicity (4–6). Biofilm was formed via several complex processes, and many virulence factors are involved in bacterial adhesion, exopolysaccharide formation, sugar uptake, and acid tolerance (7). In S. mutans, water-insoluble (α-1,3-linked) and soluble (α-1,6-linked) glucans play an important role in the biofilm formation. They were synthesized by glucosyltransferases (GTFs) from sucrose (8). Glucans not only constitute the major polymeric matrix of biofilm, which can support the structural integrity and stability of the biofilm, but also mediate S. mutans to adhere to the tooth surfaces and enhance the aggregation and coaggregation of the bacterial cells (9). Currently, dental plaque is mainly controlled by the treatment of broad-spectrum antibiotics or mechanical removal, but the effect is limited (10). Although many strategies have been settled to control biofilms, the pursuit of natural, safe, and effective antibiofilm agents continues (11, 12).

Lactobacillus species as probiotic bacteria are being used in many dairy products to provide health benefits (13). One of the beneficial effects of Lactobacillus species as probiotics is the inhibition of pathogen infection. The mechanism includes the production of antimicrobial compounds such as organic acids, H_2_O_2_, bacteriocins, and adhesive inhibitors; improvement of epithelial barrier function; and inhibition of the adhesive ability of pathogens to epithelial cells (14, 15). Lactobacillus also has been reported that exerts healthy effects on the oral cavity. For example, the culture supernatant of Lactobacillus acidophilus can attenuate gingivitis and alleviate periodontitis (16). In addition, Lactobacillus rhamnosus GG, Lactobacillus plantarum, and Lactobacillus reuteri were reported to inhibit biofilm formation of S. mutans (17). Coculture of Lactobacillus salivarius with S. mutans inhibited the biofilm formation by regulating the glucosyltransferase (*gtf*) genes expression. However, most studies focused on the inhibition of biofilm formation by using cells, and the mechanisms by which lactic acid bacteria (LAB) inhibits the formation of biofilm have not been well understood. Recently, Ahn et al. (18) reported that lipoteichoic acid, the cell wall component, isolated from L. plantarum KCTC10887BP can reduce biofilm formation of S. mutans without bactericidal activity. The structure of lipoteichoic acid was crucial for inhibitory activity.

However, few studies have investigated the mechanism of supernatants of Lactobacillus on biofilm formation by S. mutans. Although substances such as organic acids and hydrogen peroxide in the supernatant contributed to a large extent to the inhibitory effect against biofilm formation, some studies have shown that even neutralized and catalase-treated supernatant still showed inhibitory activity (19). This shows the possibility that some compounds other than acid and hydrogen peroxide may be present in the supernatant. To investigate the exometabolites produced by LAB and explore the application of exometabolites on dental caries, this study focused on the exploration of the antibiofilm compounds from the supernatant of LAB. For this aim, we isolated lactic acid bacteria from the saliva of infants and screened the ethyl acetate extract of supernatant for antibiofilm activity. As a result, two single compounds with strong antibiofilm activities were purified, and the structure analysis showed that these compounds are iminosugar compounds, the structures of which are discovered for the first time in this study. Furthermore, the inhibitory mechanism was studied in the gene levels.

## RESULTS

### Isolation and characterization of isolates from an infant’s oral cavity.

**FIG 1:**
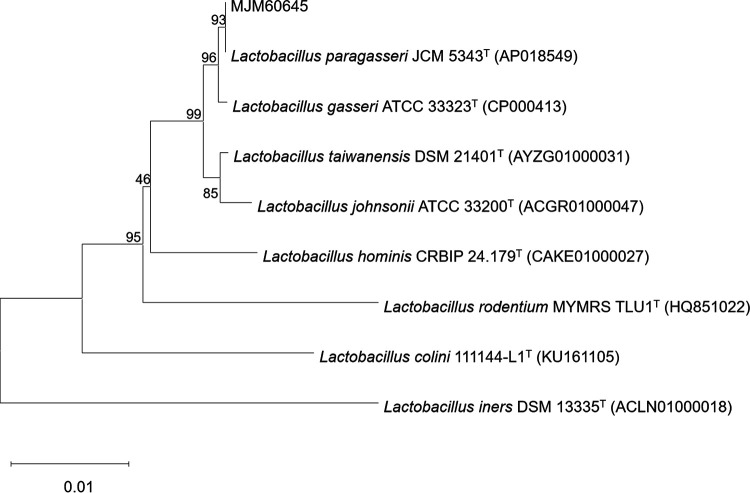
Neighbor-joining tree based on the 16S rRNA gene sequences showing the phylogenetic relationship of strain MJM60645 and its related taxa. The accession numbers in parentheses were obtained from NCBI. The numbers at nodes indicate percentage levels of bootstrap support based on a neighbor-joining method of 1,000 replications. The evolutionary distances were computed using the Kimura two-parameter method. The scale bar indicates 0.005 substitutions per nucleotide position.

A total of 11 LAB strains were isolated from the oral cavity of infants. 16S rRNA gene sequences showed that four strains were Streptococcus rubneri, three strains were Lactobacillus paragasseri, two strains were Streptococcus salivarius, and two strains were Leuconostoc mesenteroides. Among these isolates, strain MJM60645 exhibited the highest antagonistic activity, and its extract showed the strongest antibiofilm activity (Table 1). Phylogenetic analysis based on the 16S rRNA sequence exhibited that MJM60645 was more closely related to L. paragasseri JCM 5343 (99%) than to Lactobacillus gasseri ATCC 33323 (98.3%) (Fig. 1).

**TABLE 1:** The antagonistic and antibiofilm activity of lactic acid bacteria against S. mutans

Strain no.	Strain name^*a*^	Antagonistic activity (mm)	Antibiofilm activity^*b*^ (%)
MJM60645	Lactobacillus paragasseri	30	48
MJM60646	Lactobacillus paragasseri	28	42
MJM60648	Lactobacillus paragasseri	27	40
MJM60649	Streptococcus rubneri	15	2
MJM60651	Leuconostoc mesenteriodes	26	34
MJM60654	Streptococcus salivarius	25	21
MJM60655	Streptococcus salivarius	15	15
MJM60657	Leuconostoc mesenteroides	18	31
MJM60658	Streptococcus rubneri	15	8
MJM60659	Streptococcus rubneri	16	2
MJM60660	Streptococcus rubneri	15	6

a The highest similarity based on the 16S rRNA gene sequence.

b Antibiofilm activity of crude EtOAc extract at the concentration of 1 mg/mL.

### Antagonistic activity of the isolates and antibiofilm activity of the LAB crude ethyl acetate (EtOAc) extracts.

The antagonistic test showed that the L. paragasseri MJM60645 strain showed the strongest antagonistic activity against S. mutans. S. rubneri, L. mesenteroides, and S. salivarius strains showed relatively weak or moderate activity, and the inhibition zone ranged from 15 to 18 mm. However, one of the S. salivarius and one of the L. mesenteroides showed strong activities with inhibition zones greater than 25 mm in diameter.

Crude EtOAc extracts (1 mg/mL) from different LAB strains were treated with S. mutans KCTC3065, and the antibiofilm activities were assessed. Crude extract from L. paragasseri MJM60645 showed strong antibiofilm activity, extracts from L. mesenteroides showed moderate activity, and extracts from S. salivarius and S. rubneri showed moderate or low activity (Table 1).

### Stability of crude BuOH extract of L. paragasseri MJM60645.

**FIG 2:**
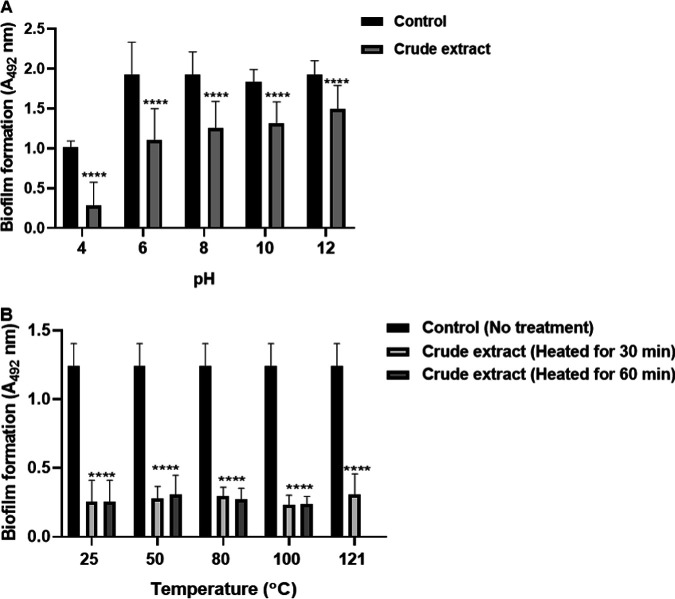
pH and thermal stability of crude extract of L. paragasseri MJM60645. (A) Biofilm formation in the presence of the crude extract of different pH values. Control was treated with water of different pH levels. (B) Biofilm formation after treated with crude extracts that were heated at different temperatures for 30 or 60 min or 121°C for 15 min. Control was not treated with the crude extract. The data are expressed as the means ± standard deviations from three biologically independent experiments. ****, *P < *0.0001, indicating a significant difference compared with the control groups. Statistical analysis was performed with one-way analysis of variance (ANOVA) with Dunnett’s multiple-comparison test.

To assess the pH and thermal stability of crude BuOH extract, samples were exposed to different pH levels (pH 4, 6, 8, 10, and 12) and different temperatures (50, 80, 100, and 121°C). It was found that the crude extract could strongly inhibit biofilm formation when treated with acidic conditions, and the activity was stronger than control. In neutral or alkali conditions, the extract of L. paragasseri MJM60645 lost activity to a certain extent but still showed activity compared with nontreated control (Fig. 2A). Interestingly, the crude extract showed significant thermal stability, and the inhibitory activity remained unchanged after treatment at different temperatures for different times, even at 121°C for 15 min (Fig. 2B).

### Effect of crude BuOH extract on the viable cell number of planktonic and biofilm.

**FIG 3:**
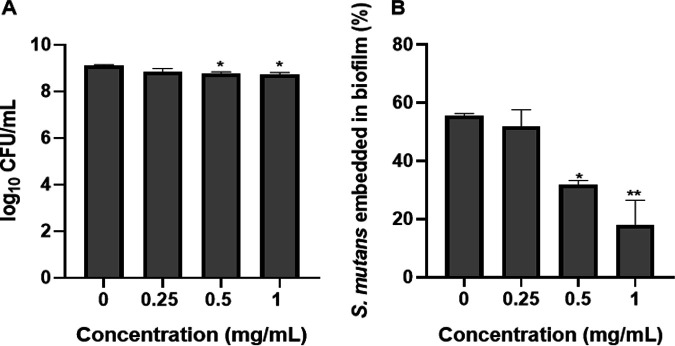
Effect of crude extract on the number of viable S. mutans in planktonic and biofilm cells. (A) The total viable S. mutans (planktonic + biofilm) after treated with the crude extracts at different concentrations. *, *P < *0.05 by Student’s two-tailed *t* test. (B) The cells embedded in the biofilm were compared to the total cells. The CFU values represent the means ± standard deviations from three independent experiments. The asterisks indicate the significant difference between control and extract-treated groups. *, *P < *0.05; **, *P < *0.01. Statistical analysis was performed with one-way ANOVA with Dunnett’s multiple-comparison test.

As shown in Fig. 3A, the effect of crude BuOH extract on the cell population was determined by the CFU of S. mutans. In the control group, the log CFU of total bacteria (planktonic + biofilm cell) is 9.1. In the treatment group, the log CFU were 8.84, 8.75, and 8.72 at the concentration of 0.25, 0.5, and 1 mg/mL, respectively. There was a 0.2 to 0.3 log unit CFU of decrease in the treatment groups compared with the control.

S. mutans embedded in the biofilm were compared to the total bacteria (planktonic + biofilm cells) and is expressed as a percentage. The crude extract inhibited the percentage of S. mutans embedded in the biofilm in a dose-dependent manner. The percentage of cell number in biofilm decreased with the increase of the concentration treated, while the percentage of the planktonic cell increased. At a concentration of 1 mg/mL, less than 20% of S. mutans were embedded in the biofilm (Fig. 3B).

### Isolation and structure identification of the active compounds.

To isolate the active compounds, organic solvent extraction and chromatography were applied. Among the 16 fractions collected from reverse-phase chromatography, fractions LPE-B-SEC6-RP7 (compound 1) and LPE-B-SEC6-RP10 (compound 2) showed a single spot in the thin-layer chromatography (TLC) analysis and strong activity in the antibiofilm assay. These two compounds have no UV absorption at 254 nm nor 365 nm but can be visualized by 10% H_2_SO_4_ staining. The structures were identified primarily via one- and two-dimensional NMR spectroscopy and high-resolution electrospray ionization-time of flight mass spectrometry (HR-ESI-TOF-MS).

**FIG 4:**
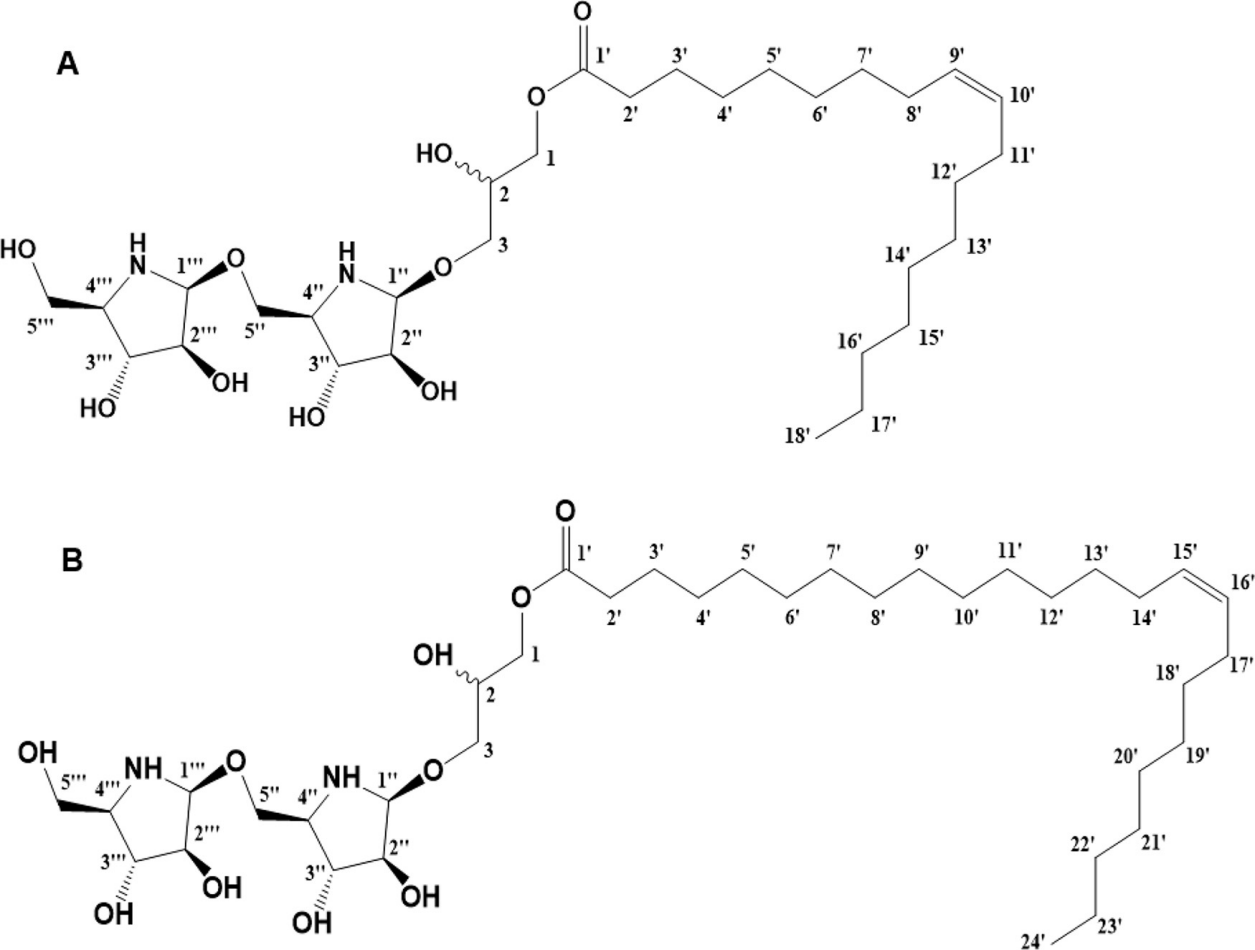
Structures of antibiofilm compounds produced by L. paragasseri MJM60645. (A) Structure of compound 1. (B) Structure of compound 2.

Compound 1 was isolated as a yellowish oil, and its molecular formula was established as C_31_H_59_N_2_O_10_ by HR-ESI-TOF-MS (*m/z* 619.4218 [M+H]^+^; calculated, 619.4164; error, 8.7 ppm). Analysis of H and C NMR spectra of compound 1 indicated that it was an iminosugar compound. In H NMR, the presence of the olefin methane proton signal was confirmed at *δ*_H_ 5.13 (2H, t-like, *J *= 4.8 Hz, H-9′,10′). The presence of two oxygenated methylene signals (*δ*_H_ 4.22 [2H, dd, *J* = 7.2, 7.2 Hz, H-1], 3.66 [2H, dd, *J* = 7.8, 7.8 Hz, H-3]) and one oxygenated methane proton signal *δ*_H_ 3.51 (1H, overlapped, H-2) indicated the presence of a molecule of glycerol. The proton at *δ*_H_ 4.22 (H-1) showed a signal at a lower field than the other oxygenated methylene proton signal due to the esterification effect caused by the combination of oleic acid. In addition, the presence of two pentoses was confirmed by the two hemiacetal proton signals (*δ*_H_ 4.47 [1H, d, *J* = 4.2 Hz, H-1″], 3.97 [1H, d, *J* = 3.5 Hz, H-1″′]), six oxygenated methine proton signals (*δ*_H_ 4.60 [1H, dd, *J *= 4.8, 4.2 Hz, H-2″], 4.07 [1H, m, H-2″′], 3.90 [1H, overlapped, H-3″], 3.89 [1H, overlapped, H-4″], 3.76 [1H, m, H-4″′], and 3.52 [1H, dd, *J *= 4.8, 4.8 Hz, H-3″′]) and two oxygenated methylene proton signals (*δ*_H_ 3.60 [2H, overlapped, H-5″], 3.60 [2H, overlapped, H-5″′]). The signal of two hemiacetal protons (*δ*_H_ 4.47 [H-1″], 3.97 [H-1″′]) was observed in a high field, indicating that the carbon in position 1 or 4 of the pentose was replaced with nitrogen. Based on the molecular weight, molecular formula, and ^1^H NMR data, compound 1 was a glycoside containing a oleic acid, a glycerol, and two nitrogen-containing arabinofuranoses (Fig. 4A).

In ^13^C NMR (150 MHz, CD_3_OD, *δ*_C_) spectrum, one ester carbon signal *δ*_C_ 175.3 (C-1′), two olefin methane carbon signals (*δ*_C_ 130.9 [d, C-9′], 130.8 [d, C-10′]), fourteen methylene carbon signals (*δ*_C_ 35.0 [C-2′], 33.1 [C-16′], 30.8 to 30.2 [C-4′-7′,12′-15′], 28.1 [C-8′], 28.1 [C-11′], 26.0 [C-3′], and 23.7 [C-17′]), and one terminal methyl carbon signal *δ*_C_ 14.5 (C-18′) were observed in a low magnetic field. Compared with the reference, these signals represented the presence of oleic acid. The signal of one oxygenated methane *δ*_C_ 71.3 (C-2) and two oxygenated methylene carbon signals (*δ*_C_ 70.1 [C-3] and 64.6 [C-1]) indicated the presence of glycerol. The third carbon signal of glycerol was observed in *δ*_C_ 70.1, showing the shift to the low field due to the glycosidation effect of the combination of glycerol with a glycoside. In addition, the presence of two hemiacetal (*δ*_C_ 87.3 [C-1″], 86.0 [C-1″′]), six oxygenated methine (*δ*_C_ 81.9 [C-2″′], 81.8 [C-2″], 74.4 [C-3″], 73.6 [C-3″′], 71.1 [C-4″], and 70.9 [C-4″′]), and two oxygenated methylene carbon signals (*δ*_C_ 70.0 [C-5″] and 62.2 [C-5″′]) indicated the presence of two pentoses. Due to the signal for hemiacetal carbon (*δ*_C_ 87.3 [C-1″] and 86.0 [C-1″′]) being observed in Highfield, we predicted that the carbon in one or four positions in pentose was replaced with nitrogen. If the carbon in position 1 was replaced with nitrogen, there should be a peak at 45 to 55 ppm according to reference 20. The absence of the peak at 45 to 55 ppm confirmed that the carbon in position 4 was replaced with nitrogen as β-d-4-amino-4-deoxy-arabinofuranose. Moreover, the shift of the carbon signal of C-5″ (*δ*_C_ 70.0) to the low field due to the glycosidation effect indicated that the two pentoses were connected as β-d-4-amino-4-deoxy-arabinofuranosyl-(1→5)-*O*-β-d-4-amino-4-deoxy-arabinofuranose.

In the gradient-selected heteronuclear multiple-bond correlation (gHMBC) spectrum, the cross-peak between the H-1 proton (*δ*_H_ 4.22) of glycerol and easter carbon (C-1′, *δ*_C_ 175.3) of oleic acid indicated the linkage of oleic acid to the oxygenated methylene of glycerol C-1′. The cross-peaks between *δ*_H_ 4.47 (H-1′′) and *δ*_C_ 70.1 (C-3), between *δ*_H_ 3.97 (H-1″′) and *δ*_C_ 70.0 (C-5″), and between *δ*_H_ 3.60 (H-5″) and *δ*_C_ 86.0 (C-1″′) indicated that β-d-arabinofuranose was linked to the C-3 of glycerol, and another β-d-arabinofuranose linked to the oxygenated methylene C-5 of β-d-arabinofuranose. Combined with the 2D NMR data and mass spectrum, compound 1 was finally identified as 1-*O*-oleoyl-3-*O*-[β-d-4-amino-4-deoxy-arabinofuranosyl-(1→5)-*O*-β-d-4-amino-4-deoxy-rabinofuranosyl]-glycerol, a new iminosugar compound (Fig. 4A).

Compound 2 was obtained as yellowish oil. Its molecular formula was deduced as C_37_H_70_N_2_O_10_ from NMR and positive-ion HR-ESI-QTOF-MS data (*m/z* 747.4981 [M+FA−H−]; calculated, 747.5013; error, 4.2 ppm). A comparison of the 1D NMR data (Table 2) of compound 2 with those of compound 1 revealed that they shared the same sugar and glycerol moiety, and the only obvious difference between them was that the fatty acid connected to the glycerol moiety is nervonic acid in compound 2, compared with oleic acid in compound 1. Compound 2 was finally identified as 1-*O*-nervonoyl-3-*O*-[β-d-4-amino-4-deoxy-arabinofuranosyl-(1→5)-*O*-β-d-4-amino-4-deoxy-rabinofuranosyl]-glycerol (Fig. 4B). The key NMR and mass spectrum are presented in supplementary data (Fig. S2 to S13), and the detailed ^1^ H and ^13^C assignments are given in Table 2.

**TABLE 2:** ^1^ H (600 MHz) and ^13^C (150 MHz) NMR data for compounds 1 and 2 in CD_3_OD (CD_3_OH for NH protons)^*a*^

No.	Compound 1		Compound 2	
	*δ*_H_	*δ*_C_	*δ*_H_	*δ*_C_
1	4.22 (dd, 7.2, 7.2)	64.6	4.17 (d, 4.8)	64.6
2	3.51 (overlapped)	71.3	3.51 (overlapped）	71.3
3	3.66 (dd, 7.8, 7.8)	70.1	3.66 (dd, 5.6, 5.6)	70.2
1′		175.3		175.4
2′	2.30 (t, 7.2)	35.0	2.30 (t, 7.2)	35.0
3′	1.58 (t-like, 7.2)	26.0	1.58 (t-like, 7.2)	26
4′	1.27 (overlapped)	30.8–30.2	1.26 (overlapped)	30.8–30.1
5′	1.27 (overlapped)	30.8–30.2	1.26 (overlapped)	30.8–30.1
6′	1.27 (overlapped)	30.8–30.2	1.26 (overlapped)	30.8–30.1
7′	1.27 (overlapped)	30.8–30.2	1.26 (overlapped)	30.8–30.1
8′	2.00 (overlapped)	28.1	1.26 (overlapped)	30.8–30.1
9′	5.13 (t-like, 4.8)	130.9	1.26 (overlapped)	30.8–30.1
10′	5.13 (t-like, 4.8)	130.8	1.26 (overlapped)	30.8–30.1
11′	2.00 (overlapped)	28.1	1.26 (overlapped)	30.8-30.1
12′	1.27 (overlapped)	30.8–30.2	1.26 (overlapped)	30.8-30.1
13′	1.27 (overlapped)	30.8–30.2		30.8-30.1
14′	1.27 (overlapped)	30.8–30.2	2.00 (overlapped)	28.1
15′	1.27 (overlapped)	30.8–30.2	5.31 (m)	130.9
16′	1.27 (overlapped)	33.1	5.31 (m)	130.8
17′	1.27 (overlapped)	23.7	2.00 (overlapped)	28.1
18′	0.87 (t, 7.2)	14.5	1.26 (overlapped)	30.8–30.1
19′			1.26 (overlapped)	30.8–30.1
20′			1.26 (overlapped)	30.8–30.1
21′			1.26 (overlapped)	30.8–30.1
22′			1.26 (overlapped)	33.1
23′			1.26 (overlapped)	23.7
24′			0.87 (t, 6.6)	14.5
1″	4.47 (d, 4.2)	87.3	4.47 (d, 3.6)	87.3
2″	4.60 (dd, 4.8, 4.2)	81.8	4.6 (dd, 3.6, 3.6)	81.8
3″	3.90 (overlapped)	74.4	3.89 (overlapped)	74.4
4″	3.89 (overlapped)	71.1	3.86 (overlapped)	71.1
5″	3.6 (overlapped)	70.0	3.6 (overlapped)	70.0
1‴	3.97 (d, 3.5)	86.0	3.96 (br·s)	86.0
2‴	4.07 (m)	81.9	4.07 (m)	81.9
3‴	3.52 (dd, 4.8, 4.8)	73.6	3.52 (dd, 5.6, 5.6)	73.6
4‴	3.76 (m)	70.9	3.77 (m)	70.9
5‴	3.60 (overlapped)	62.2	3.60 (overlapped)	62.2

a NMR, nuclear magnetic resonance.

### Determination of IC_50_ values of compounds 1 and 2.

**FIG 5:**
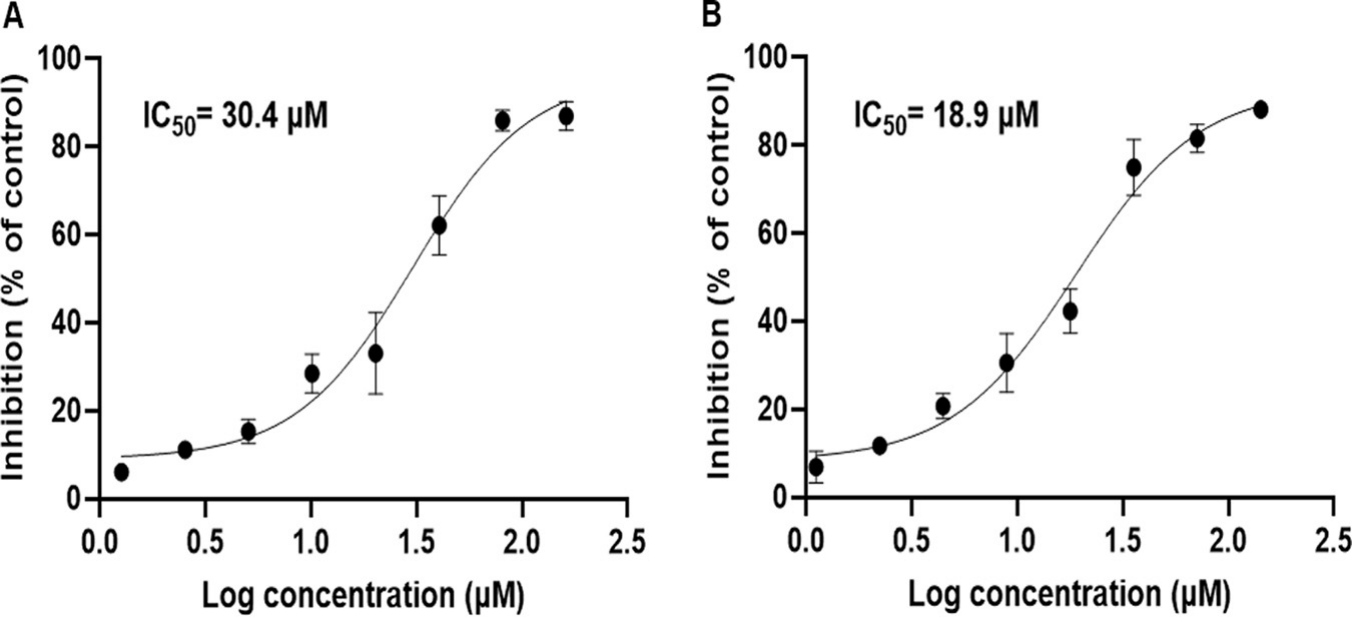
Determination of antibiofilm IC_50_ of isolated compounds. (A, B) Dose-response curve of inhibition by compounds 1 (A) and 2 (B) on S. mutans biofilm formation. The data are presented as mean percentages of inhibition ± standard deviation for a total of five samples (pooled from three independent experiments in triplicate). The percentage of inhibition is relative to phosphate-buffered saline (PBS) control.

The concentration that inhibits S. mutans biofilm formation by 50% (IC_50_) for compounds 1 and 2 were determined by serial dilution. S. mutans were treated with compound 1 or 2 at a concentration ranging from 1 to 160 μM. It was found that biofilm formation was dose-dependently inhibited by either compound 1 or 2. The IC_50_ values were determined to be 30.42 and 18.95 μM for compounds 1 and 2, respectively (Fig. 5A and B).

### Effect of compounds 1 and 2 on early and mature biofilm formation of S. mutans.

**FIG 6:**
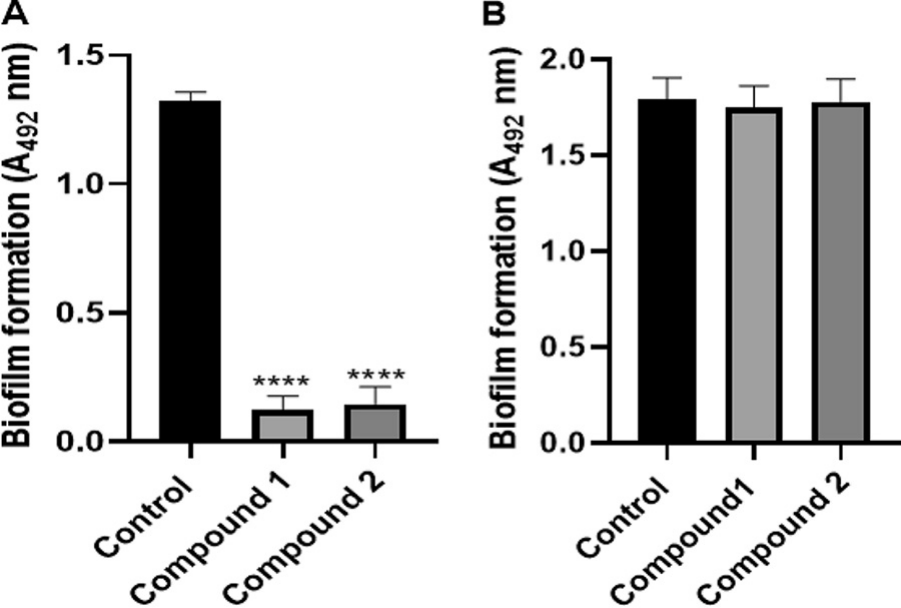
Effect of compounds 1 and 2 on early (A) and mature (B) biofilm formation. (A) S. mutans was treated with compounds 1 and 2 at the concentration of 60 and 36 μM, respectively, at the beginning of the assay. After incubation for 24 h, the biofilm was measured. (B) S. mutans was incubated in a brain heart infusion (BHI) medium containing 0.2% sucrose in a 96-well plate. After incubation for 24 h, the developed biofilm was treated with compounds 1 and 2 and incubated for a further 24 h. The biofilm was determined as described above. All measurements were performed in triplicates, and the mean values ± SD were calculated. ****, *P* < 0.0001. Statistically significant differences were calculated by one-way ANOVA with Dunnett’s multiple-comparison test.

When the active compounds were treated at the beginning of the biofilm assay, they showed significant inhibitory activities to the biofilm formation of S. mutans. Both compound 1 (treated at 60 μM) and compound 2 (treated at 38 μM) inhibited more than 90% of biofilm (Fig. 6A). However, there was no significant difference between the control and treated groups when the active compounds were treated to the preformed biofilm and incubated for a further 24 h (Fig. 6B).

### Expression of the biofilm-associated gene of S. mutans after treatment with compound 1.

**FIG 7:**
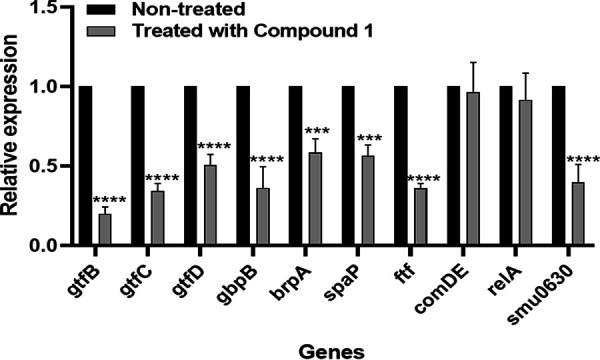
Effect of compound 1 on the expression of biofilm-associated genes in S. mutans. S. mutans was treated with compound 1 at the concentration of 60 μM in a BHI medium containing 0.2% of sucrose and incubated anaerobically at 37°C for 24 h. Biofilm cells were harvested, and the expression of biofilm-associated genes was assessed by real-time quantitative PCR (qRT-PCR). The mRNA expression levels were calibrated using 16S rRNA. The data are expressed as the means ± standard deviations from three biologically independent experiments. The asterisks indicate that the gene expression between the control group (white bars) and the compound 1-treated group (black bars) was significantly different. *, *P < *0.05; **, *P < *0.01; ***, *P < *0.001. Statistical analysis was performed with one-way ANOVA with Dunnett’s multiple-comparison test.

Since compound 2 was purified in trace amounts, we evaluated the effect of compound 1 on the expression of biofilm-associated genes. Relative expression levels of selected genes were examined by real-time quantitative PCR (qRT-PCR). Compared with the control group, the expression levels of eight biofilm-associated genes (*gtfB*, *gtfC*, *gtfD*, *gbpB*, *brpA*, *spaP*, *ftf*, and *smu0630*) were significantly downregulated by compound 1. However, expression levels of *comDE* and *relA* genes in S. mutans showed no significant difference between the treatment group and the control group (Fig. 7).

## DISCUSSION

Oral microbiota is constituted of a large number of microorganisms; dominant among them are Firmicutes, Proteobacteria, Bacteroidetes, Actinobacteria, Fusobacteria, etc. (21). These bacteria are closely related to oral health. Among them, S. mutans is considered a primary causative agent of dental caries, and one of the virulence properties of this bacteria was to form a biofilm known as dental plaque on the tooth surface (22). On the other hand, one of the beneficial bacteria that live in the oral cavity is lactic acid bacteria, like S. salivarius and Lactobacillus spp. LAB was reported to inhibit the biofilm formation of S. mutans either by coculture (23) or by treatment of the culture supernatant to the S. mutans (24). Even though the use of Lactobacillus strains for probiotic applications has been carried out recently (25), the mechanism for the interaction between Lactobacillus and S. mutans remains largely unknown. In this study, we screened the EtOAc extract of different LAB strains for the antibiofilm activity and isolated two iminosu gar compounds from the culture supernatant of L. paragasseri MJM60645. To the best of our knowledge, this is the first report on the small molecule with strong antibiofilm activity isolated from the supernatant of Lactobacillus spp. Furthermore, the structures of these compounds were discovered for the first time through this study.

It was reported that Lactobacillus species, especially L. gasseri, were isolated more frequently from the saliva of breastfed infants than from formula-fed infants and displayed probiotic traits *in vitro* (26). Furthermore, Lactobacillus strains from caries-free subjects possess antimicrobial activity against the cariogenic species of S. mutans and Streptococcus sobrinus, and the activity was more efficient than those from caries-active subjects (27). Therefore, this study was initiated based on the hypothesis that Lactobacillus from caries-free infants may play a beneficial effect on oral health. In this study, four species of LAB strains were isolated from the infant’s saliva in the MRS medium, including S. salivarius and S. rubneri from a 30-month-old child, L. mesenteroides from a 10-month-old baby, and L. paragasseri from a 1-month-old infant. S. salivarius and S. rubneri are commonly isolated from the human cavity and upper respiratory tract (28, 29), and some strains of S. salivarius were used as a probiotic for improving oral health (28). L. mesenteroides is typically found on the surface of many fresh fruits and vegetables and is associated with fermentation. Intake of fermentative food, fruits, and vegetables by a 10-month-old baby may be the reason for the existence of L. mesenteroides in the oral cavity. L. gasseri is a widely studied probiotic species with various biological functions, including oral probiotic characteristics (30). L. gasseri is known as a part of the vaginal flora, and some researchers isolated L. gasseri from breast milk (31) or saliva (27). Recently, many of L. gasseri were reclassified to L. paragasseri due to the distinct difference based on whole-genome relatedness, phylogenetic analyses of the marker gene, and phenotypic and chemotaxonomic characteristics (32). According to the phylogenetic analysis of the 16S rRNA gene sequence, strain MJM60645, along with L. paragasseri JCM4353, did form a clade distinct from L. gasseri ATCC 33323^T^ (Fig. 1). Thus, strain MJM60645 was finally designated L. paragasseri MJM60645.

The first screening was carried out by evaluation of their potential antagonistic activities against S. mutans. Antagonistic activity against pathogens is an important characteristic in the selection of potential probiotic strains for maintaining a healthy microbial balance in the oro-gastrointestinal tract. In the present study, all isolates tested showed antagonistic activities against S. mutans, but the activities are various. Strains in the same species showed different activities, indicating that antagonistic activity is strain-specific, not species-specific. Among these strains, L. paragasseri MJM60645 showed the highest antagonistic effect. To investigate the extracellular metabolite produced by LAB, the supernatant of the culture supernatant from each LAB was extracted with ethyl acetate, a widely used organic solvent for extraction of biological compounds with various polarities from water solution. All of these extracts did not show the antimicrobial activity by the paper disc agar diffusion assay (data not shown). However, in the test for antibiofilm activity, these extracts showed inhibitory activity to the biofilm formation of S. mutans at different levels. Among them, the extract from L. paragasseri strains showed stronger activity than other extracts. Since these L. paragasseri strains were isolated from the same donor and showed similar activities, we speculated that these isolates were the same or were genetically related. Thus, one of the L. paragasseri, MJM60645, was selected for further study.

Before purification, the stability of crude extract from L. paragasseri MJM60645 was investigated. The crude extract treated in alkali and neutral conditions showed decreased activities, but extracts in acidic conditions showed strong activity. We later found that the pH of compound 1 in the water solution was acidic, which is why the crude extract lose most of its activity in neutral or alkali conditions. Although the control (water in pH 4) also showed inhibitory activity, the activity was weaker than the extract-treated group. This indicated that acidity was not the only reason for the inhibitory activity. The extract works well in acidic conditions, which will help to inhibit S. mutans because S. mutans make an acidic environment on the tooth surface to erode the tooth through acid production. In addition, the extract showed strong thermal stability under different temperatures, even under 121°C, indicating that the active compound is probably a small molecule rather than a macromolecular protein.

The total number of planktonic and biofilm cells was compared between the crude BuOH extract-treated and untreated groups. There was a 0.2 to 0.3 log CFU reduction in the total number of bacteria after being treated with BuOH extract, suggesting that the extract affected S. mutans but not a lot. This may be due to the growth rate or biofilm formation because the BuOH extract did not show any bactericidal activity by agar diffusion assay (data not shown). Interestingly, the ratio of bacteria embedded in biofilm was dose-dependently decreased by the treatment of crude BuOH extract, indicating that the extract influenced the formation of the biofilm, allowing more planktonic bacteria.

To purify the active compounds, the ethyl acetate extract of L. paragasseri supernatant was partitioned with several organic solvents and applied to size exclusion chromatography and open column reverse-phase chromatography (Fig. S1). The activity of these subfractions was monitored by an antibiofilm assay. Finally, two single compounds (compounds 1 and 2) were obtained. The structures were chemically characterized by ^1^H NMR (one- and two-dimensional) and ^13^C NMR, and the molecular weights were analyzed by high-resolution mass spectrometry (Fig. S2 to S13). The structure of compound 1 is composed of two iminosugars (arabinofuranose in which the cyclic oxygen is replaced with nitrogen), one molecule of glycerol, and one molecule of oleic acid, which were linearly connected by a hydrogen bond. Compound 2 is a structural analog of compound 1, in which oleic acid was replaced by nervonic acid (Fig. 4A and B). The novelty of the structures was confirmed on the SciFinder database.

Iminosugars are structural analogs of carbohydrates in which the oxygen atom is replaced by nitrogen. The first iminosugar, 1-deoxynojirimycin (DNJ), was isolated from mulberry in 1976, and a few others were discovered recently (33). Due to the structural similarity to the sugar, iminosugar compounds showed strong catalytic properties to ubiquitous carbohydrate processing enzymes, such as glycosidases and glycosyltransferase, exhibiting broad-spectrum therapeutic potential in the treatment of type II diabetes, lysosomal storage disorders, etc. Furthermore, the iminosugar class of compounds was known to show antiviral activity by inhibiting the endoplasmic reticulum (ER) α-glucosidase activity due to the structural similarity to sugar molecules. Moreover, some researchers have investigated the effect of iminosugars on oral biofilms. It was reported that iminosugars can affect the biosynthesis of glucan; it might be good for caries prevention (34). Another recent study has showed that 1-deoxynojirimycin (from mulberry leaves) significantly inhibited the biofilm formation of S. mutans (4, 35). We also found that compounds 1 and 2 could significantly inhibit biofilm formation when treated at an early stage but cannot inhibit mature biofilm (Fig. 6). This indicates that their antibiofilm mechanism might be based on the reduction of exopolysaccharides synthesis in the early stage of biofilm formation. Similarly, some iminosugars can also decrease the biofilm formation of P. aeruginosa at an early stage of biofilm development but not in the mature biofilm stage (36). Our results were consistent with the previous report about iminosugars, meaning that the antibiofilm effect of the two new compounds might be due to the presence of iminosugar moieties in their structures.

Compounds 1 and 2 have structural differences only in the moiety of fatty acids, like oleic acid in compound 1 and nervonic acid in compound 2. Although the structure of iminosugar may play a major role in the activity, the difference in MIC suggests that fatty acid moiety may also affect the magnitude of the activity. Recent studies have shown that fatty acids (FAs) possess good antibiofilm activities. Some FAs were reported to inhibit biofilm formation of various pathogens (37, 38). Oleic acid (*cis*-9-octadecenoic acid) can inhibit initial adhesion and biofilm development by some S. aureus strains (39, 40). However, it has been reported that treatment of oleic acid did not inhibit the biofilm formation of S. mutans (41). When the fatty acids (saturated and unsaturated) were incorporated into S. aureus through a FAs kinase-dependent pathway, the membrane fluidity will be changed (42, 43). Even though the fatty acid may not inhibit biofilm directly, it may affect the efficacy of the incorporation of compounds 1 and 2 into the cell, hence affecting the activities.

To adapt to a biofilm lifestyle, biofilm cells of S. mutans experience phenotypic changes, accompanied by a series of genes that are up- or downregulated. Our studies revealed that compound 1 downregulated eight biofilm-associated genes in S. mutans. These genes included glucosyltransferase genes *gtfb*, *gtfc*, and *gtfd* (44, 45), adherence-associated gene *spaP*, biofilm regulatory protein *brpA*, glucan-binding protein *gbpB* (46), fructosyltransferase *ftf* gene, and hypothetical protein *smu630*. Glucosyltransferases *gtfB* and *gtfC* can utilize sucrose as a substrate to synthesize water-insoluble glucan, which is necessary for backbone construction and structural integrity of biofilms. Gene *gtfd* synthesizes α-1,6-linked soluble glucan. It is reported that the alkali-soluble glucans are relatively more important than water-soluble glucan in bacterial adhesion (47). 1-Deoxynojirimycin (DNJ) was reported to inhibit glucosyltransferases B rather than glucosyltransferases C and D (48). Our result also showed that compound 1 inhibited *gtfb* more strongly than *gtfc* and *gtfd* (Fig. 7). The *ftf* gene encodes fructosyltransferase that can synthesize fructan polymers, which provides binding sites for S. mutans, thus enhancing its biofilm formation (49). The suppression of *gtf* genes may be associated with reduced biofilm formation. In addition, some other genes involved in bacterial attachment to the tooth surface and biofilm structure integrity were also inhibited. Glucan-binding protein (coded by *gbpB*) has an affinity for glucans, which resulted in bacterial adhesion (46). Gene *spaP* (Ag I/II or P1) encodes a surface protein of the antigen, which is important for S. mutans to initially adhere to the tooth surface (50). Gene *brpA* (biofilm regulatory protein) is involved in biofilm development and its structural integrity. *Smu630*, a hypothetical protein, also contributes to biofilm formation (51). Downregulation of the expression of these genes can suppress glucan formation and cell adhesion, thus reducing biofilm formation. However, the expression of other biofilm-associated genes is not affected, including the *relA* gene that encodes guanosine tetra (penta)-phosphate synthetase, which is involved in the mechanisms of acid and oxidative stress tolerance in S. mutans (52). The regulatory gene *comDE*, involving the quorum-sensing cascade of S. mutans (53), was also not affected by the treatment of compound 1 (Fig. 7). These results demonstrated that compound 1 inhibited biofilm mainly by downregulation of the expression of genes that are associated with cell adherence and biofilm development.

Currently, the selection of natural compounds seems to bring a new perspective to the development of antibiofilm compounds that can effectively inhibit the formation of exopolysaccharide chains, therefore increasing the permeability of biofilms to antibiotics, disinfectants, or nano compounds. In this study, we discovered two iminosugar compounds with novel structures from L. paragasseri MJM60645. These compounds showed strong antibiofilm activity but not antimicrobial activity. This could facilitate the use of these compounds in removing biofilms without worrying about antibiotic resistance. Undoubtedly, more studies are needed to demonstrate the molecular mechanism of the inhibitory activity of active compounds and define the specificity and antibiofilm spectrum of these compounds against various bacterial species. It is also necessary for further study to investigate the synergic effect of these compounds when combined with antibiotics for the treatment of biofilm-forming clinical pathogens.

Another significance of this study is that we provide new clues about the interactions of oral microbes. It is necessary to investigate the production of these compounds or their analogs by other microbes in future work, and the inhibitory mechanism should be deeply elucidated. Furthermore, a study on the biosynthetic pathway of this novel iminosugar compounds will facilitate the discovery and development of more derivatives, which can be widely used for research and the medical field.

## MATERIALS AND METHODS

### Sample collection and bacteria isolation.

Saliva samples were collected from three children aged from 1 month to 30 months. The number of erupted teeth per donor was 0 for a 1-month-old baby, 8 for a 10-month-old baby, and 20 for a 30-month-old child. All three children were caries-free. They had not taken any antibiotics in the previous 14 days. We explained the purpose of the study to the children’s parents, and they signed a consent form prior to saliva collection. The protocol for saliva collection was approved by the Institutional Review Board of Kyung Hee University (institutional review board approval KHSIRB-17-010). Briefly, a sterile cotton swab was put into the mouth of the child and rubbed two or three times until the cotton head was soaked with saliva. The cotton head was then suspended in a Falcon tube containing 3 mL of sterile saline (0.85% NaCl), stored at 4°C, and immediately taken to the laboratory.

The sample was serially diluted with sterile saline and plated onto de Man-Rogosa-Sharpe (MRS) agar plates. These plates were incubated at 37°C for 48 h under anaerobic conditions in GasPak containing an anaerobe gas-generating pouch system (BD). After incubation, colonies with different morphologies were picked for further characterization.

### Screening of antagonistic isolates.

Antagonistic activities of isolates against S. mutans KCTC3065 were assessed using an agar diffusion assay by Cadirci and Citak (17). S. mutans KCTC3065 was incubated in brain heart infusion (BHI) broth at 37°C for 24 h. The suspension of S. mutans was diluted with BHI broth medium to a turbidity equivalent to optical density at 600 nm (OD_600_) of 1. The melted BHI soft agar medium (0.7% agar) held at 45°C was then mixed with 0.1% (vol/vol) of S. mutans suspension, and 2 mL of the mixture was poured onto an MRS agar (1.5% agar) plate to make a top-covered test plate. A small hole was made on the top-covered test plate, and 5 μL of bacterial suspension of each isolate (OD_600_ = 1) was loaded into the hole. Inhibition zones were measured in millimeters after incubation at 37°C for 24 h.

### Phylogenetic analysis.

Genomic DNA was extracted and purified using the Exgene cell SV kit (GeneAll, South Korea). 16S rRNA gene was amplified using the universal primer set 27F/1492R (54) and sequenced. The resulting 16S rRNA gene sequence (1.5 kb) was blasted with available 16S rRNA gene sequences in the Ezbiocloud server (ChunLab Inc., South Korea). 16S rRNA gene sequences of closely related type strains were multiple aligned using CLUSTAL_X program (55). The phylogenetic tree was constructed with a neighbor-joining method and the Kimura two-parameter model using the MEGA 6 program (56). The robustness of individual branches was evaluated by bootstrapping of 1,000 replications.

### Preparation of crude extracts.

The isolates were inoculated in 50 mL of MRS medium and cultured at 37°C for 24 h. The culture broth was harvested and centrifuged at 7,000 rpm for 15 min. The cell-free supernatant was extracted with the same volume of ethyl acetate (EtOAc), and the ethyl acetate layer was concentrated under a vacuum to yield brownish-yellow extracts. For further purification, the EtOAc extract was dissolved in H_2_O and partitioned with hexane and then with butanol (BuOH) to get a crude BuOH extract (Fig. S1).

### Biofilm inhibitory assay.

Biofilm formation was assessed using the protocol as described previously (57) with a few modifications. Briefly, S. mutans KCTC3065 was cultured in BHI medium overnight and adjusted to an OD_600_ of approximately 1 followed by a 100-fold dilution using BHI medium supplemented with 0.2% sucrose. Then 20 μL of S. mutans suspension, 160 μL of BHI with 0.2% sucrose, and 20 μL of the crude EtOAc extract (10 mg/mL in sterile distilled water) were added to a polystyrene 96-well culture plate and incubated at 37°C for 24 h. MRS broth or phosphate-buffered saline (PBS) only served as controls. After the incubation, media and unattached cells were decanted from the microtiter plate. The remaining planktonic cells were removed by gentle rinsing with sterile distilled water. To determine the mass of biofilm, wells with adhered biofilm were stained with 125 μL of 0.1% safranin for 30 min at room temperature, washed three times with 200 μL of distilled water, and air-dried. The dye was extracted in 125 μL of 33% acetic acid, and the absorbance was measured at 492 nm by a microtiter plate reader (Tecan, Infinite M200PRO, Austria).

### pH and thermal stability of crude extract.

The crude BuOH extract was dissolved in water at a concentration of 10 mg/mL. The samples were adjusted to different pH levels (pH 4, 6, 8, 10, and 12) using HCl or NaOH and used for the inhibition of biofilm of S. mutans as described above. The distilled water was also adjusted to the same pH (4, 6, 8, 10, or 12) and served as a control group.

To evaluate the thermal stability, the crude BuOH extract was treated with different temperatures at 50, 80, and 100°C for 30 or 60 min and 121°C for 15 min. The samples were cooled down to room temperature and used for antibiofilm activity as described above.

### Effect of crude extract on the viable cell number of planktonic and biofilm.

The viable cell number of S. mutans in planktonic and biofilm were determined by serial dilution and counting of the CFU on BHI agar. Briefly, S. mutans was grown at 37°C for 24 h in glass tubes at an angle of 30° with the 3 mL of BHI broth containing 1% sucrose and various concentrations of crude BuOH extract. After incubation, the planktonic cell suspension was taken, and the cell number was determined by serial dilution and spread on the BHI agar plate. To determine the cell number in the biofilm, biofilm was scraped from the bottom of the tube and mechanically disrupted by vigorous pipetting and vortexing. These cells were serially diluted and streaked onto BHI plates. The number of colonies was then counted to determine the CFU. The percentage of S. mutans embedded in biofilm was calculated as follows: number of biofilm cells/(number of planktonic cells + number of biofilm cells) × 100%

### Fermentation, purification, and identification of active compounds from the supernatant of L. paragasseri MJM60645.

L. paragasseri MJM60645 was grown in 10 mL MRS broth for 18 h at 37°C. For fermentation, 5 mL of L. paragasseri MJM60645 suspension was inoculated to each sterile flask containing 500 mL MRS broth and incubated at 37°C for 24 h. After incubation, the supernatant of L. paragasseri MJM60645 were obtained by centrifugation at 7,000 rpm for 15 min.

An activity-guided fractionation was carried out to isolate the active compounds. Each fraction was subjected to antibiofilm bioassay and thin-layer chromatography (TLC) stained with H_2_SO_4_ (10%). Briefly, a total of 23 L of cell-free supernatant was extracted with ethyl acetate (1:1, vol/vol) and concentrated under a vacuum at 35°C. The *L. paragasseri* EtOAC extract (LPE) was partitioned by hexane and butanol step by step and concentrated under a vacuum. The active fraction from the butanol layer (LPE-B) was subjected to size exclusion chromatography (SEC) packed with Sephadex LH-20 (GE Healthcare, Uppsala, Sweden) and eluted with methanol. Among 30 fractions obtained, the most active fraction, LPE-B-SEC6 (335 mg), was subjected to reverse-phase open column chromatography (φ 2.5 × 5 cm) packed with Lichroprep RP-18 (40 to 63 μM; Merck, Darmstadt, Germany) and eluted with a stepwise gradient of acetone/water (65:35, 80:20, and 100:0) to obtain 16 subfractions (LPE-B-SEC6-RP1 to RP16). After bioassay, fractions LPE-B-SEC6-RP7 (compound 1) and LPE-B-SEC6-RP10 (compound 2) showing the strongest antibiofilm activities were finally used for the structure identification.

Chemical structures of purified compounds were analyzed by nuclear magnetic resonance (NMR, one- or two-dimensional) and mass spectrometry. NMR data were acquired with a Bruker Avance III-600 MHz recorded in CD_3_OD. HR-ESI-QTOF-MS spectra were obtained on a Bruker MaXis quadrupole time of flight (QTOF) mass spectrometer.

### Determination of IC_50_ values of compounds 1 and 2.

The IC_50_ (concentration at which 50% biofilm was inhibited) values of compounds 1 and 2 were evaluated as described previously (58). Briefly, 180 μL of BHI medium containing 0.2% sucrose was treated with serially diluted compound 1 or 2, inoculated with 20 μL of S. mutans (OD_600_ = 0.01), and incubated at 37°C for 24 h. The final concentration of each tested compound ranged from 1 to 160 μM. Biofilm assessment was carried out as described above. Bacteria treated with 20 μL of sterile distilled water were used as control.

### Effect of compounds 1 and 2 on early and mature biofilm formation of S. mutans.

To determine the effect of compounds 1 and 2 on the early biofilm formation, compounds 1 and 2 were treated to S. mutans at the concentration of 60 and 38 μM in a BHI medium containing 0.2% sucrose in a 96-well plate as described above. The plates were incubated at 37°C for 24 h, and the biofilm was quantified. To determine whether the active compounds could disperse preformed biofilms, bacterial biofilms were established for 24 h in a 96-well plate and then treated with compounds 1 and 2 at the concentration of 60 and 38 μM. The plate was incubated for an additional 24 h, and the biofilm was quantified. S. mutans treated with PBS served as the control.

### The effect of compound 1 on biofilm-associated gene expression.

The effect of compound 1 on gene expression was determined as described (49) with slight modification. The overnight culture of S. mutans was diluted to OD_600_ of 0.01 with BHI medium supplemented with 0.2% sucrose. S. mutans suspension was transferred to a 6-well plate and treated with compound 1 at the concentration of 60 μM. Only PBS served as control. The 6-well plate was incubated anaerobically at 37°C for 24 h. After incubation, the culture suspension was discarded. The cells adhering to the plate wells were gently rinsed twice with PBS, then dislodged, and suspended in saline by scraping and pipetting. Total RNA was isolated with a TaKaRa MiniBEST universal RNA extraction kit (TaKaRa Bio, Shiga, Japan) according to the manufacturer’s instructions. RNA concentration and purity were detected by the Infinite M200PRO microplate reader (Tecan, Austria). RNA (1 μg) was used to synthesize cDNA after removing the remaining genomic DNA in RNA samples by using PrimeScript RT reagent kit with gDNA eraser (TaKaRa Bio Inc., Shiga, Japan).

Quantitative real-time reverse transcription-PCR (qRT-PCR) was performed using a LightCycler 96 system (Roche, Basel, Switzerland). Each reaction mixture (20 μL) contained 100 ng of cDNA, 0.5 μM each primer, and 10 μL of SYBR Premix *Ex Taq* (TaKaRa, Japan). The qRT-PCR conditions were as follows: one cycle with 95°C for 2 min and then 40 cycles of denaturation at 95°C for 10 s, annealing, and extension at 57°C for 60 s. In all qRT-PCR runs, the negative controls without templates were run in parallel. The 16S rRNA gene, a housekeeping gene, was used as an internal control. The relative mRNA levels of genes of interest were determined and normalized to the expression of the housekeeping gene using the 2^−ΔΔ^*^CT^* method (59). Sequences of primers for qRT-PCR are listed in Table 3.

**TABLE 3:** Sequences of primers used for qRT-PCR

Genes	Primer sequence (5′ to 3′)		*T_m_* (°C)	Amplicon size (bp)	References
*ftf*	Forward	AAATATGAAGGCGGCTACAACG	56	101	50
	Reverse	CTTCACCAGTCTTAGCATCCTGAA			
*gtfB*	Forward	AGCAATGCAGCCAATCTACAAAT	58	96	50
	Reverse	ACGAACTTTGCCGTTATTGTCA			
*gtfC*	Forward	GGTTTAACGTCAAAATTAGCTGTATTAGC	59	91	50
	Reverse	CTCAACCAACCGCCACTGTT			
*brpA*	Forward	CGTGAGGTCATCAGCAAGGTC	59	148	55
	Reverse	CGCTGTACCCCAAAAGTTTAGG			
*gbpB*	Forward	CGTGTTTCGGCTATTCGTGAAG	60	108	55
	Reverse	TGCTGCTTGATTTTCTTGTTGC			
*comDE*	Forward	ACAATTCCTTGAGTTCCATCCAAG	57	81	50
	Reverse	TGGTCTGCTGCCTGTTGC			
*spaP*	Forward	GACTTTGGTAATGGTTATGCATCAA	56	101	50
	Reverse	TTTGTATCAGCCGGATCAAGTG			
*relA*	Forward	ACAAAAAGGGTATCGTCCGTACAT	58	101	10
	Reverse	AATCACGCTTGGTATTGCTAATTG			
*smu0630*	Forward	GTTAGTTCTGGTTTTGACCGCAAT	58	101	10
	Reverse	CCCTCAACAACAACATCAAAGGT			
*16S*	Forward	CCTACGGGAGGCAGCAGTAG	57	247	50
	Reverse	CAACAGAGCTTTACGATCCGAAA			

### Statistical analysis.

The experimental results were analyzed for statistical significance using GraphPad Prism (GraphPad, San Diego, CA). The results of biofilm formation quantification in the presence of crude extract, cell growth, IC_50_, and gene expression were subjected to a one-way analysis of variance (ANOVA). Data comparisons were performed using Dunnett’s multiple-comparison test. *P* values < 0.05 were considered statistically significant.

### Data availability.

The raw data used to support the findings of this study will be made available by the authors, without undue reservation, to any qualified researcher.
