# Supplementary material for: Discovery of Novel Iminosugar Compounds Produced by Lactobacillus paragasseri MJM60645 and Their Anti-Biofilm Activity against Streptococcus mutans
Source: Microbiol Spectr. 2022 Jul 6;10(4):e01122-22. doi: 10.1128/spectrum.01122-22 (PMC9431463; doi:10.1128/spectrum.01122-22)

Supplementary data

Figure S1 Flow chart of the isolation procedure.
Figure S2 ^{1}H NMR (600 MHz) spectrum of compound 1 in CD_3OD
Figure S3 ^{13}C NMR (150 MHz) spectrum of compound 1 in CD_3OD
Figure S4 gHSQC spectrum of compound 1 in CD_3OD
Figure S5 gHMBC spectrum of compound 1 in CD_3OD
Figure S6 COSY spectrum of compound 1 in CD_3OD
Figure S7 HR-ESI-QTOF-MS Mass spectrum of compound 1
Figure S8 ^{1}H NMR (600 MHz) spectrum of compound 2 in in CD_3OD
Figure S9 ^{13}C NMR (150 MHz) spectrum of compound 2 in CD_3OD
Figure S10 gHSQC spectrum of compound 2 in CD_3OD
Figure S11 gHMBC spectrum of compound 2 in CD_3OD
Figure S12 COSY spectrum of compound 2 in CD_3OD
Figure S13 HR-ESI-QTOF-MS Mass spectrum of compound 2

Figure S1

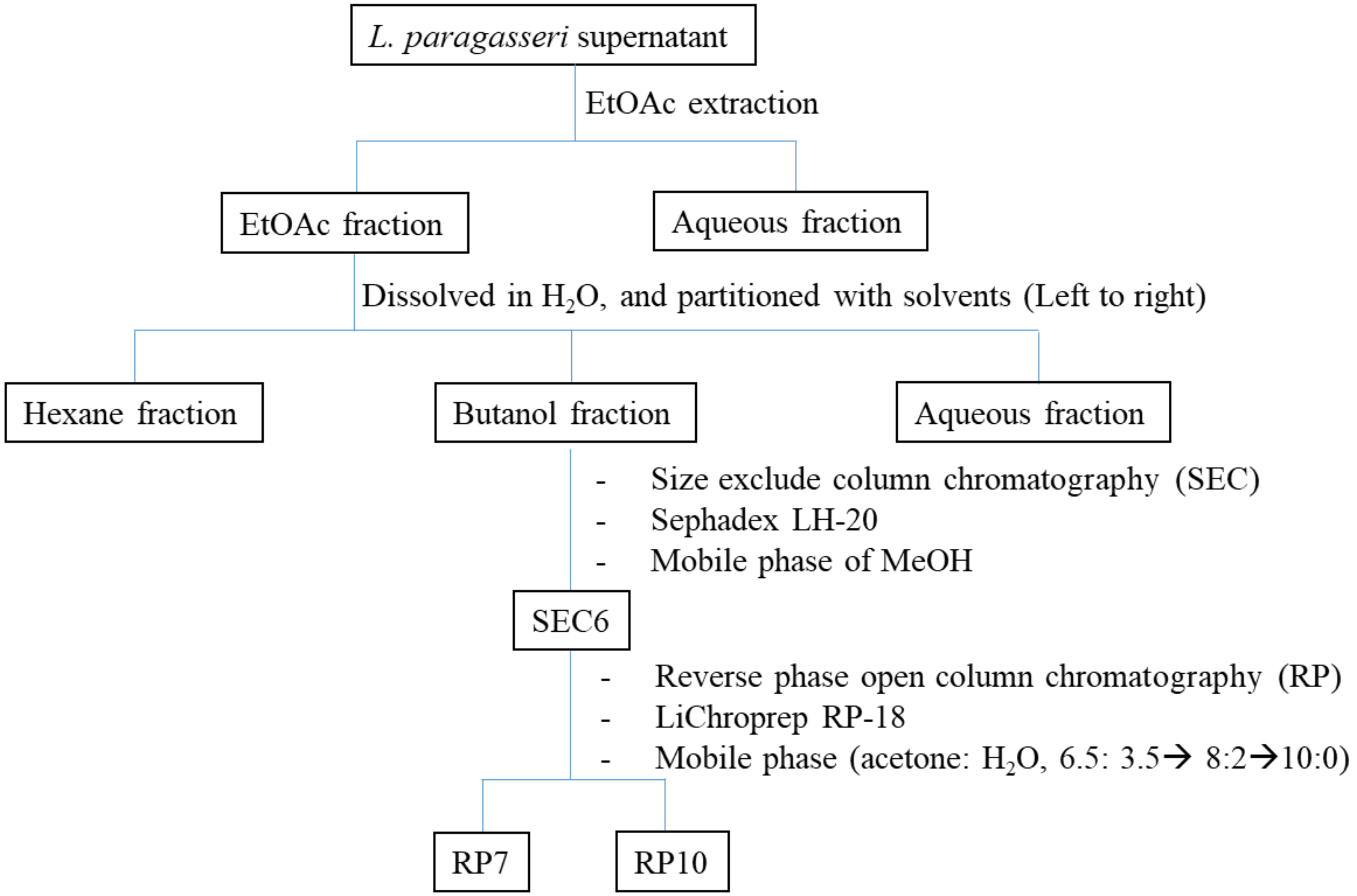
L. paragasseri supernatant
EtOAc extraction
EtOAc fraction
Aqueous fraction
Dissolved in H_2O, and partitioned with solvents (Left to right)
Hexane fraction
Butanol fraction
Aqueous fraction
- Size exclude column chromatography (SEC)
- Sephadex LH-20
- Mobile phase of MeOH
SEC6
- Reverse phase open column chromatography (RP)
- LiChroprep RP-18
- Mobile phase (acetone: H_2O, 6.5: 3.5→ 8:2→10:0)
RP7
RP10

Figure S2 ^{1}H NMR (600 MHz) spectrum of compound 1 in CD_3OD

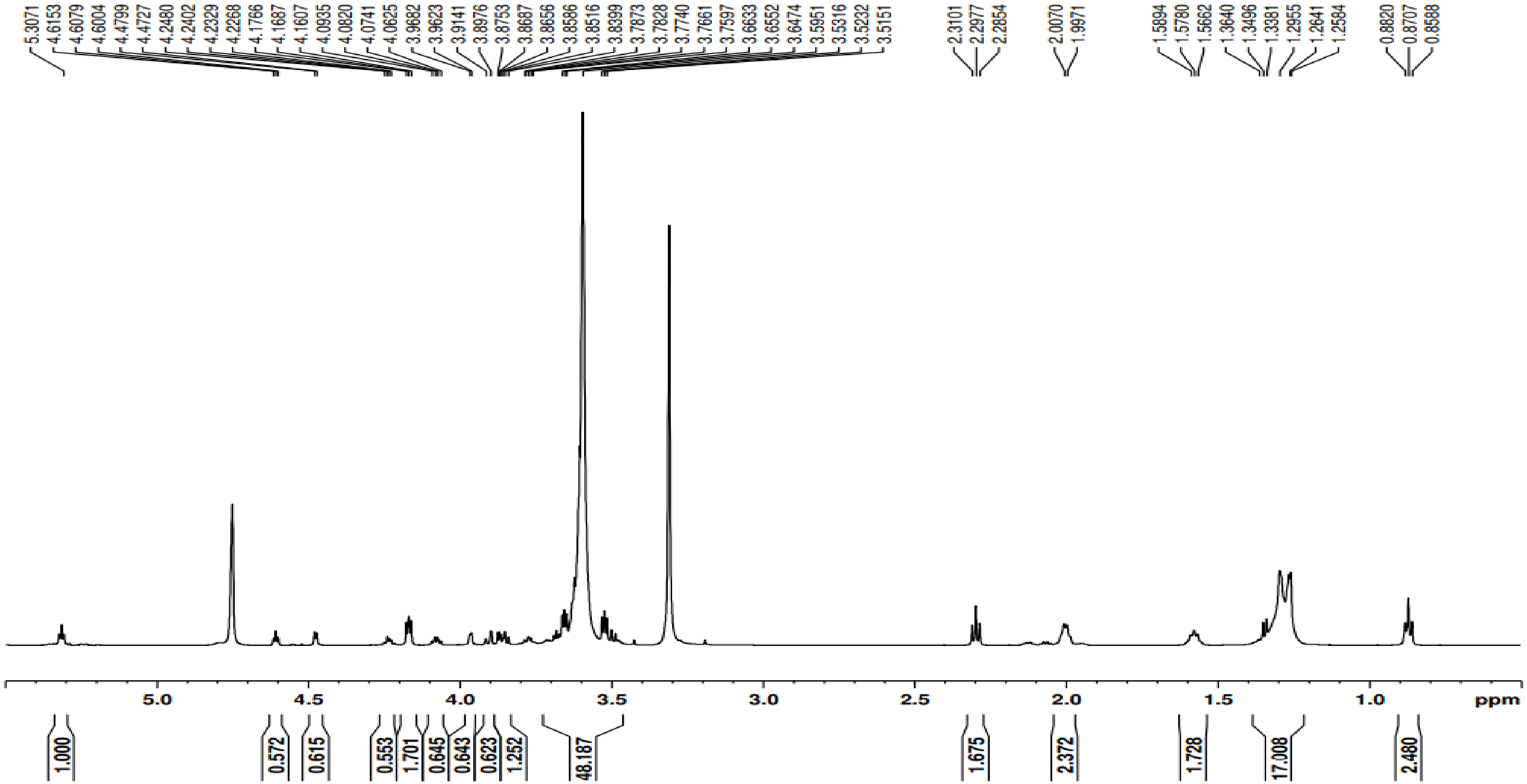

Figure S3 ^{13}C NMR (150 MHz) spectrum of compound 1 in CD_3OD

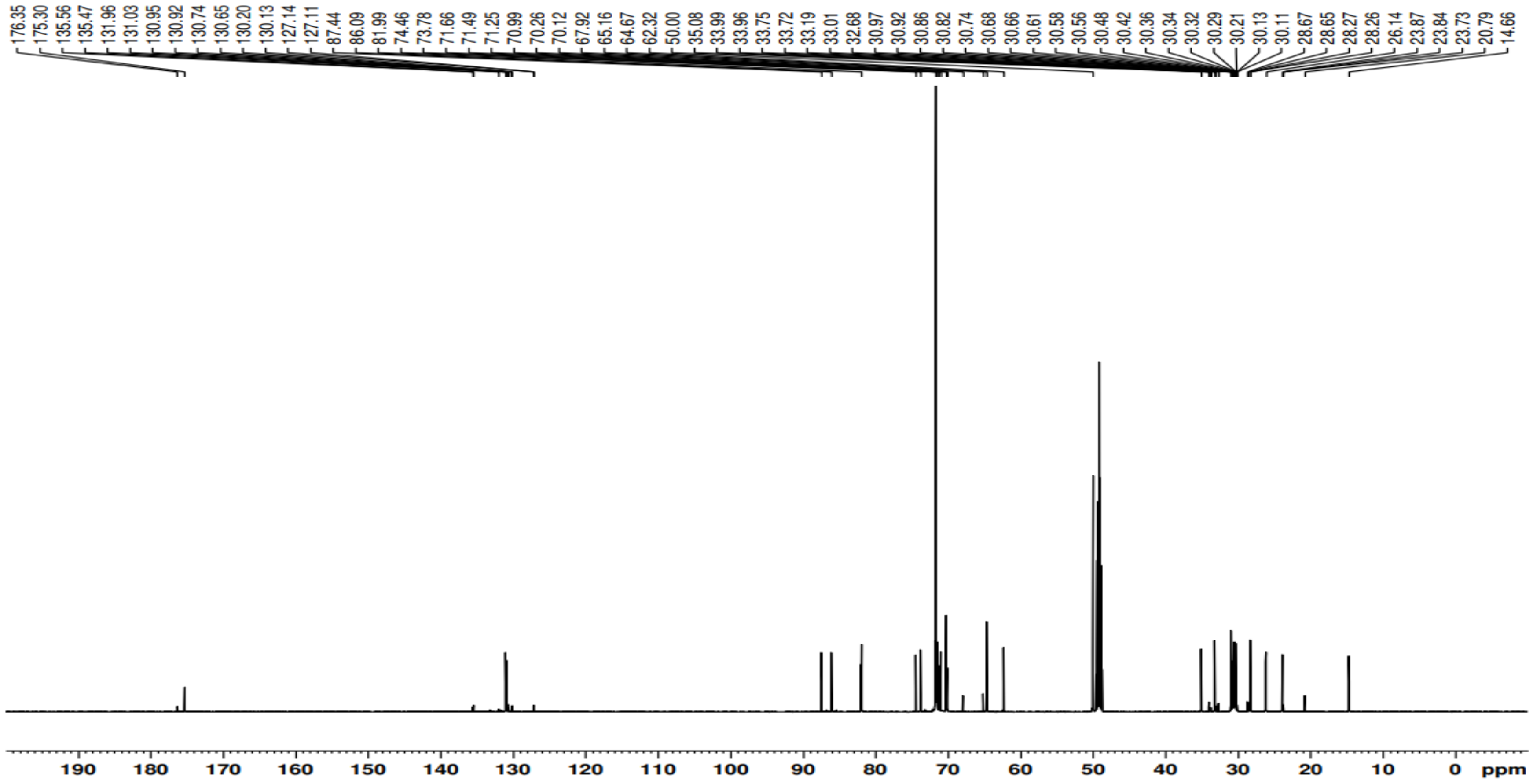

Figure S4 gHSQC spectrum of compound 1 in CD_3OD

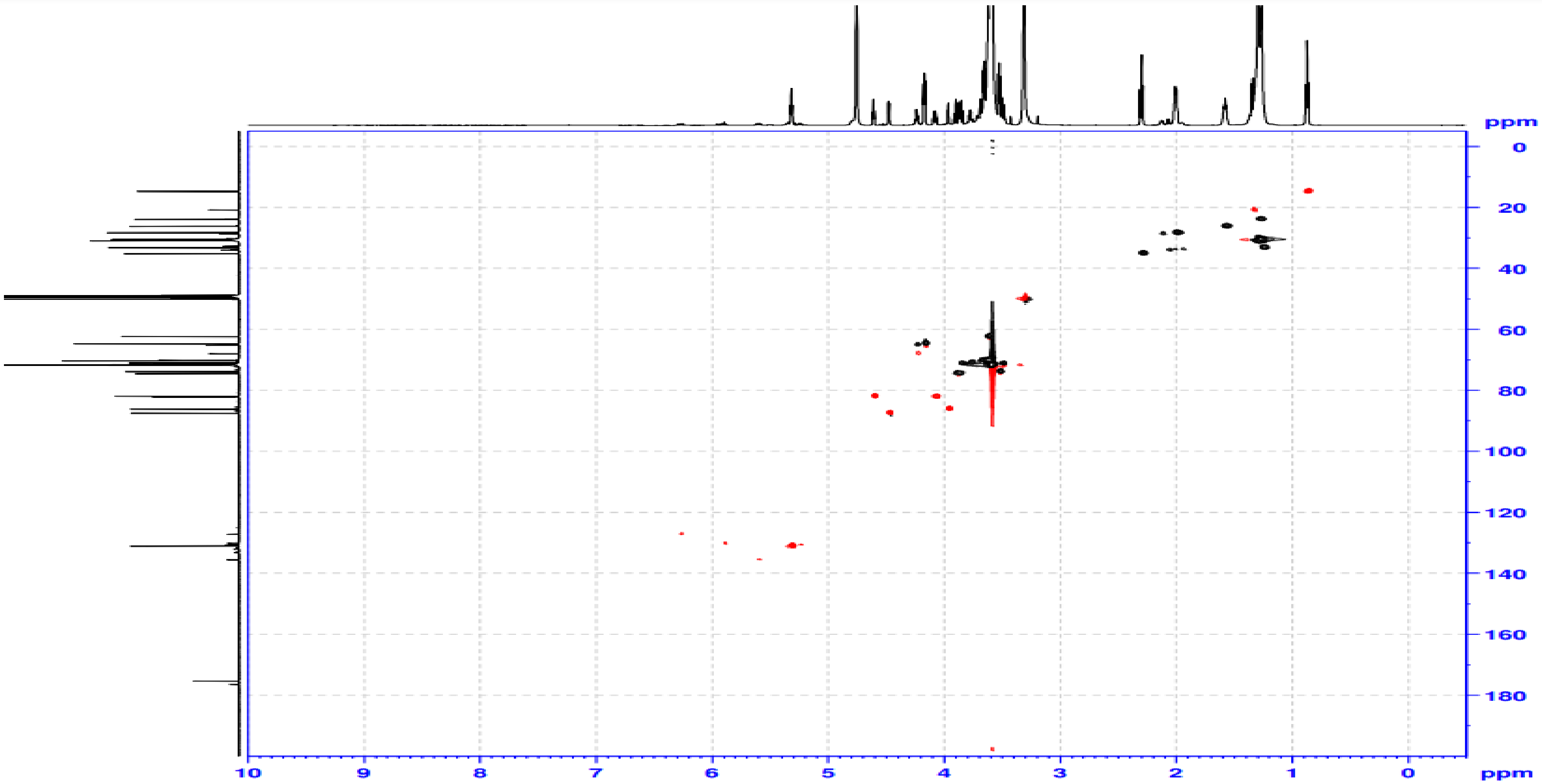

Figure S5 HMBC spectrum of compound 1 in CD_3OD

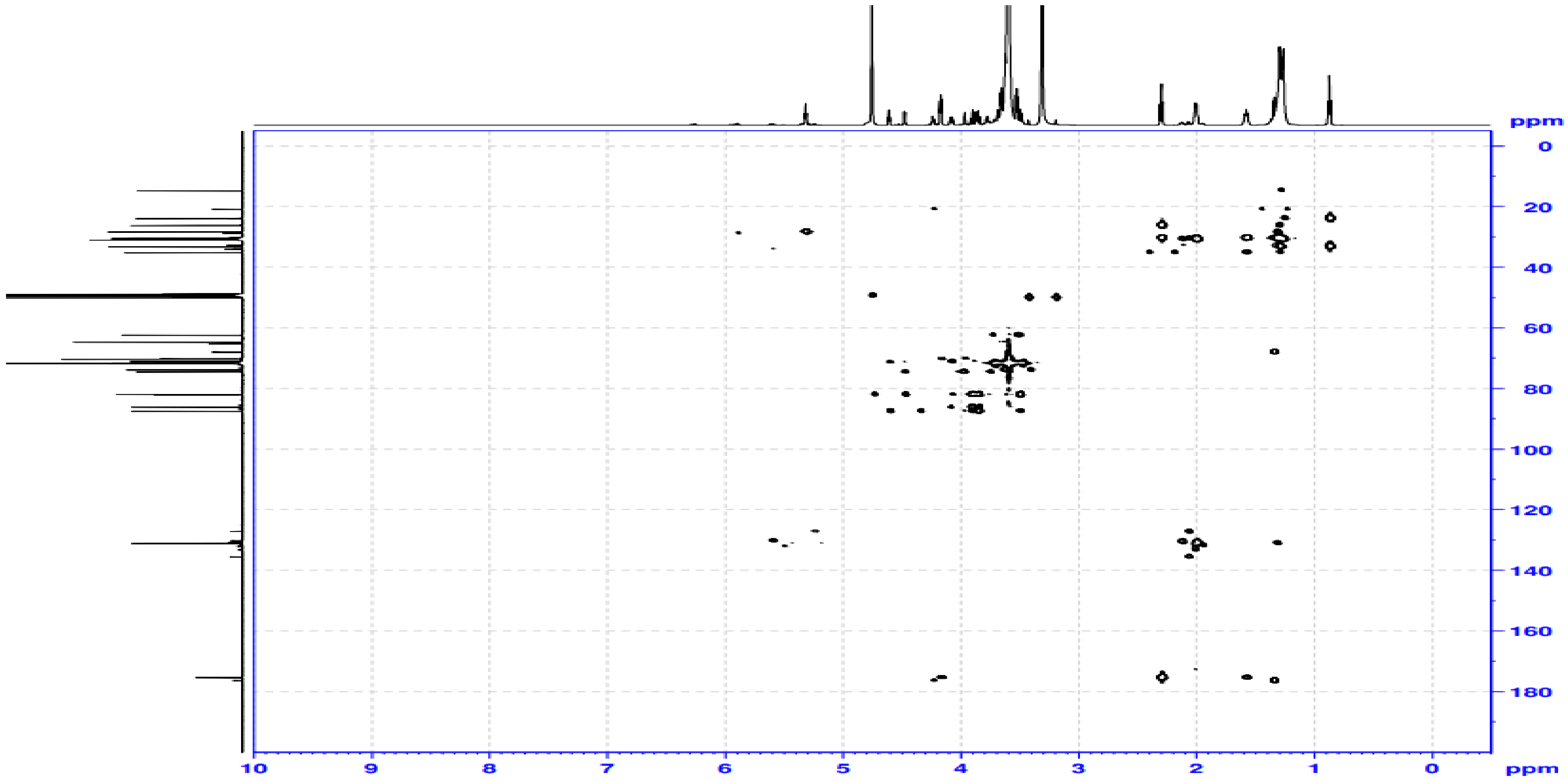

Figure S6 COSY spectrum of compound 1 in CD_3OD

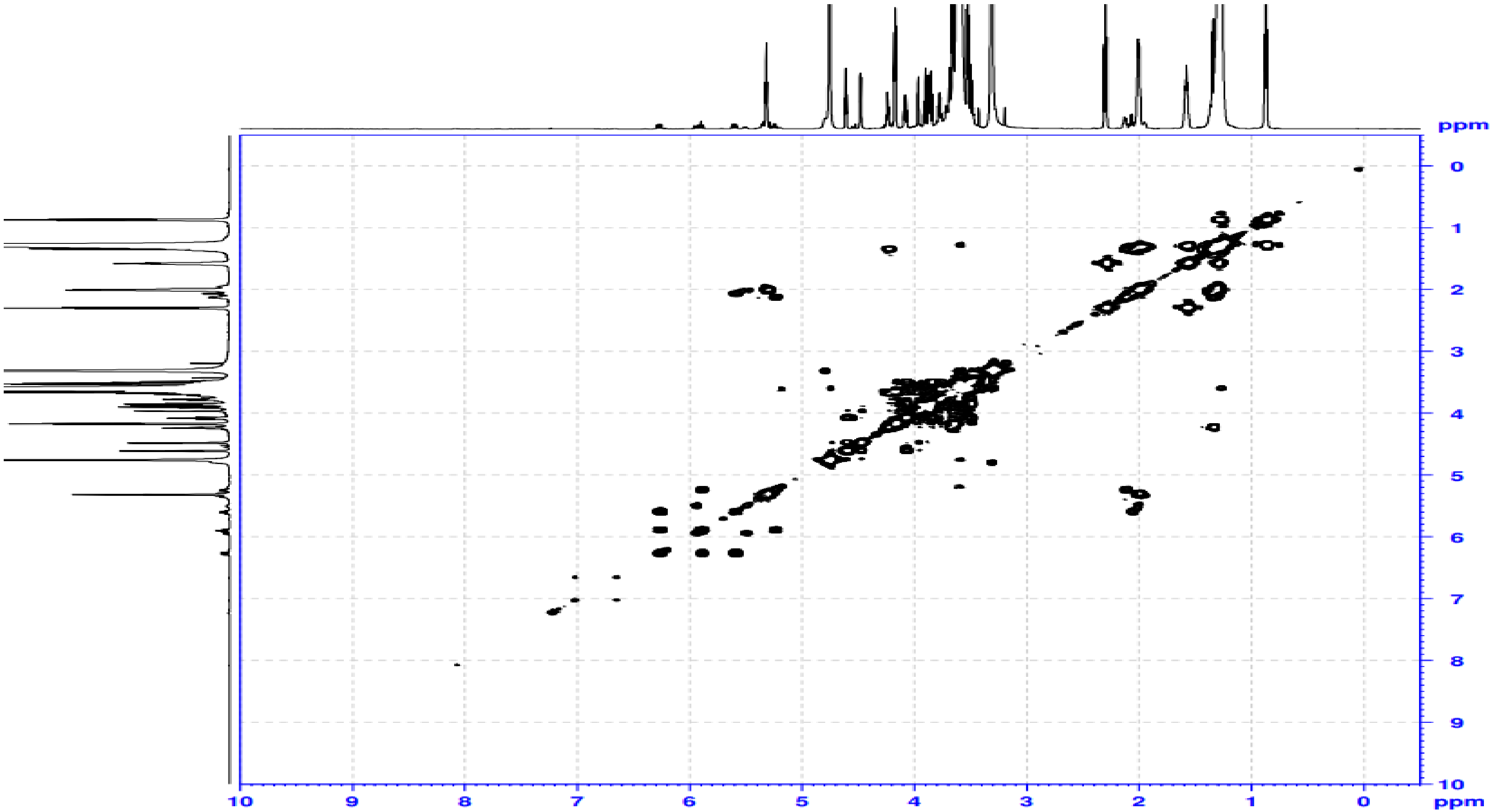

Figure S7 HR-FAB/MS spectrum of compound 1 in in CD_3OD

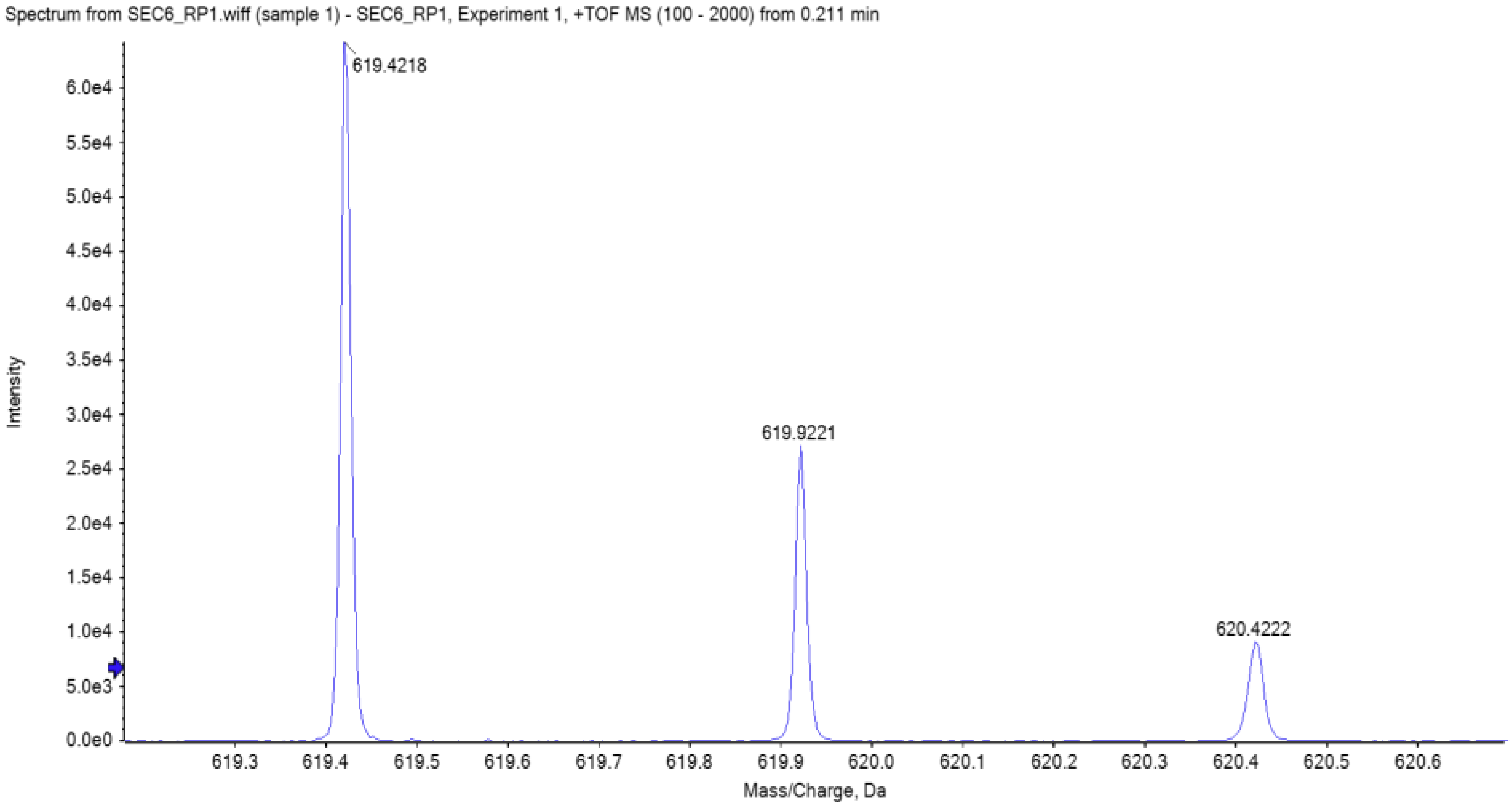

Figure S8 ^{1}H NMR (600 MHz) spectrum of compound 2 in in CD_3OD

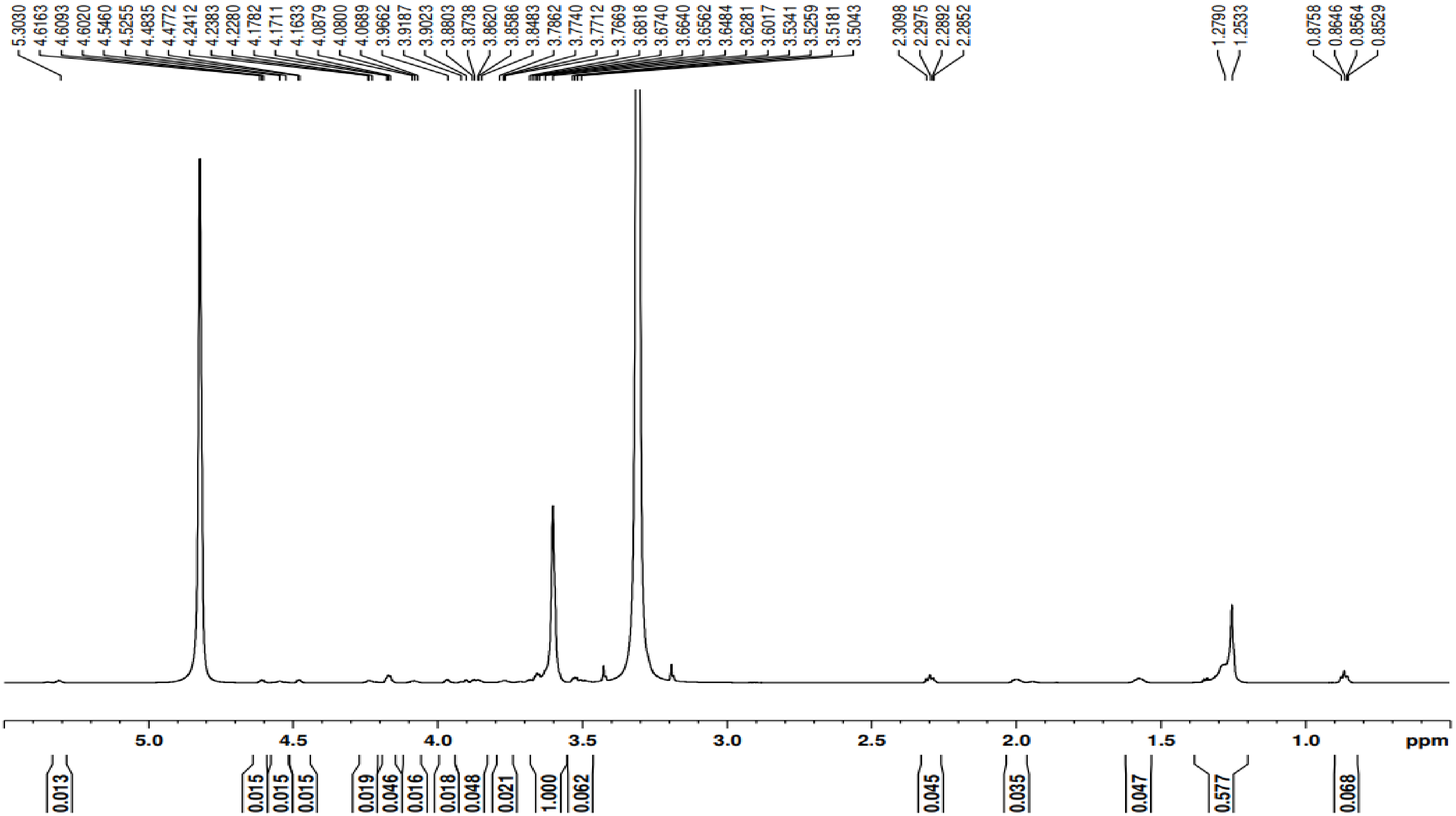

Figure S9 ^{13}C NMR (150 MHz) spectrum of compound 2 in CD_3OD

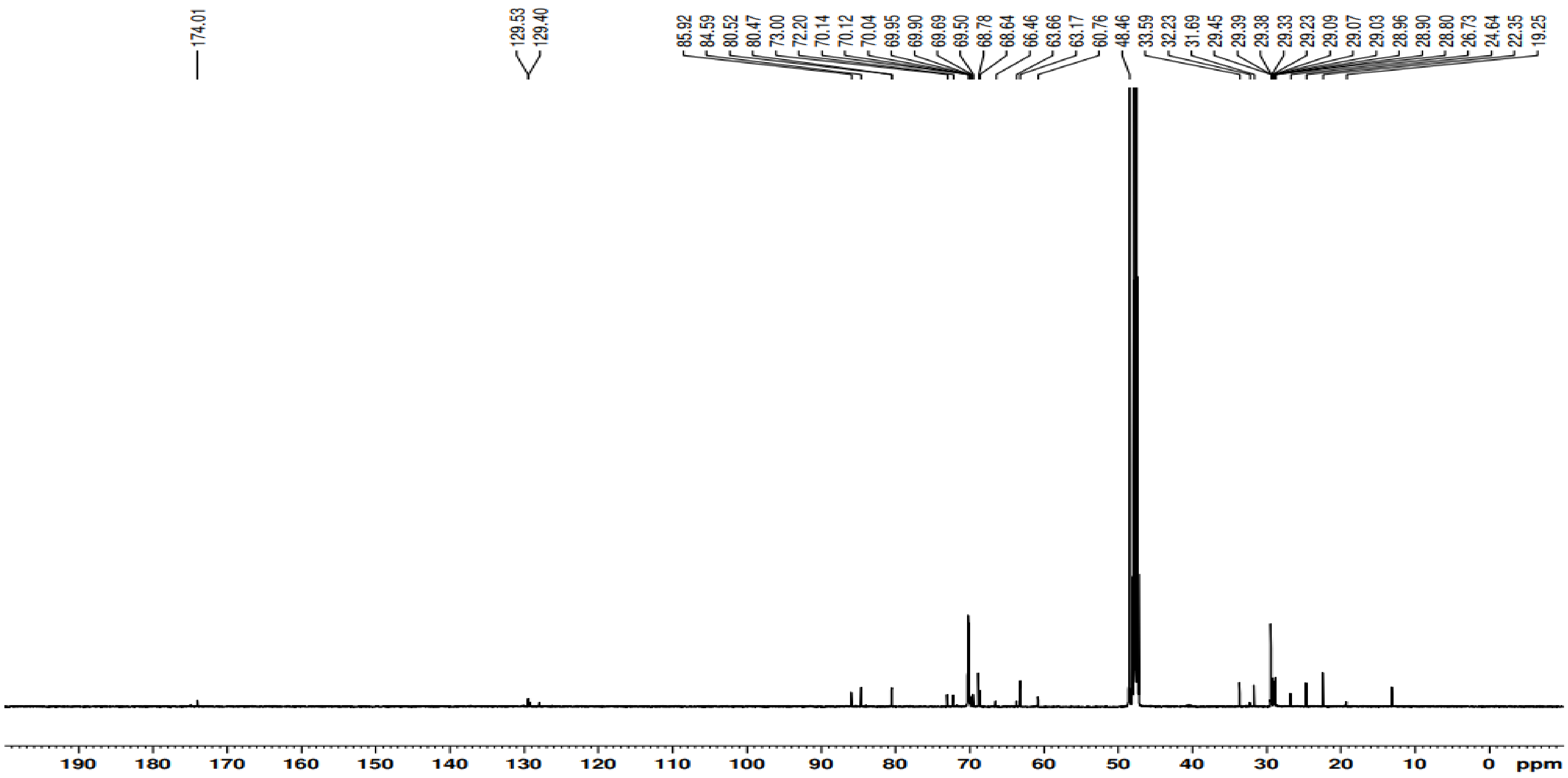

Figure S10 gHSQC spectrum of compound 2 in CD_3OD

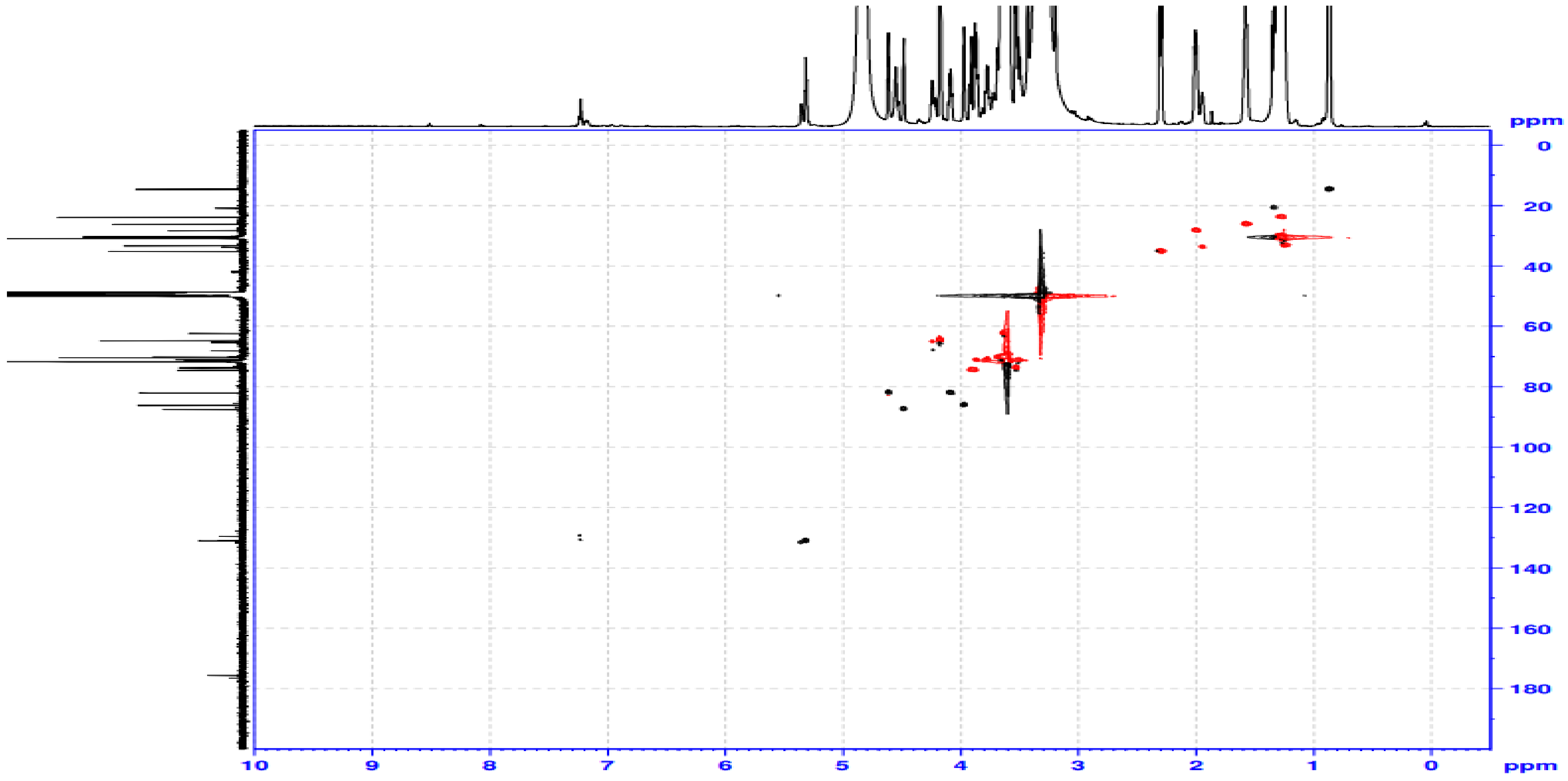

Figure S11 gHMBC spectrum of compound 2 in CD_3OD

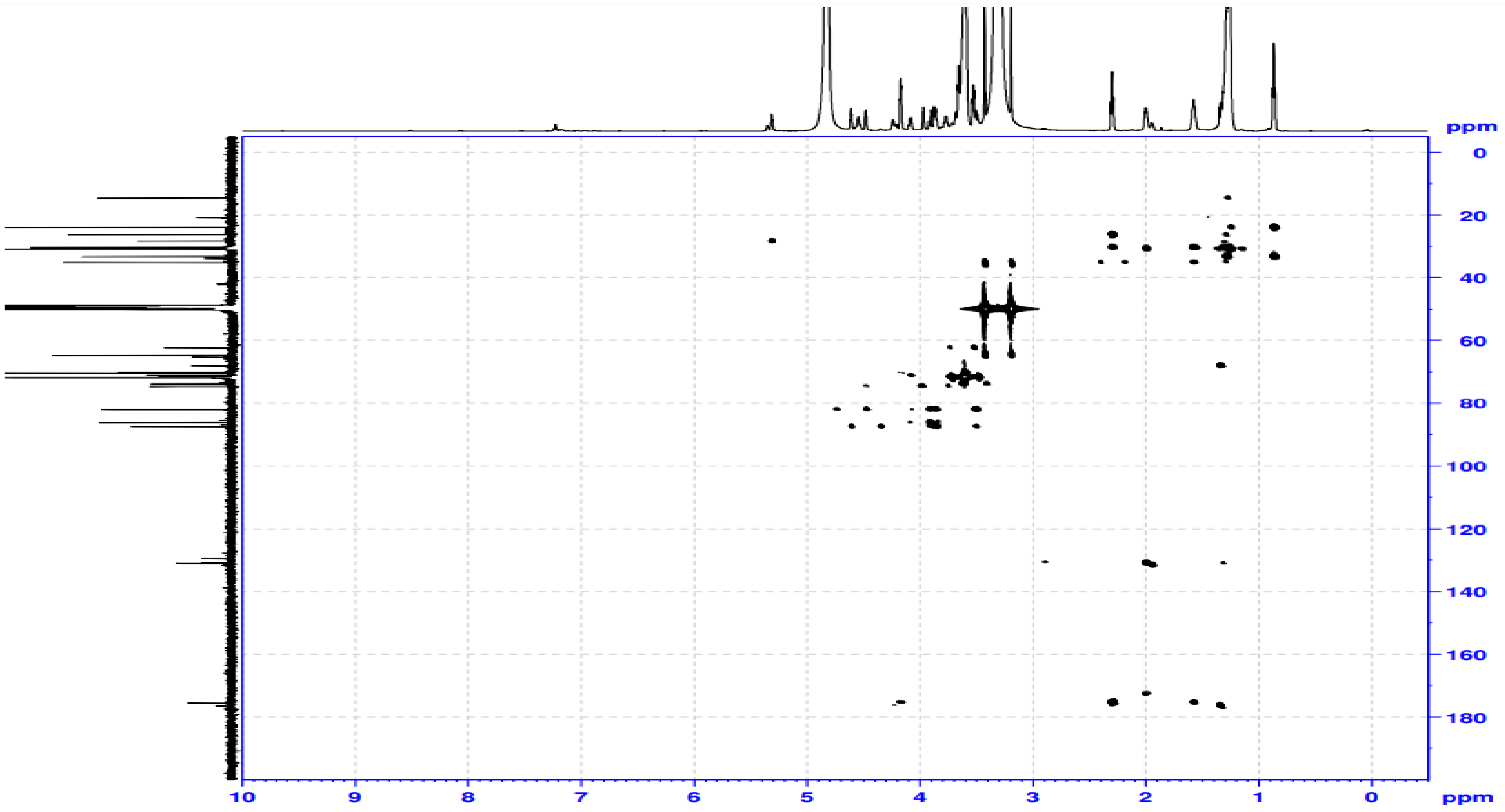

Figure S12 COSY spectrum of compound 2 in CD_3OD

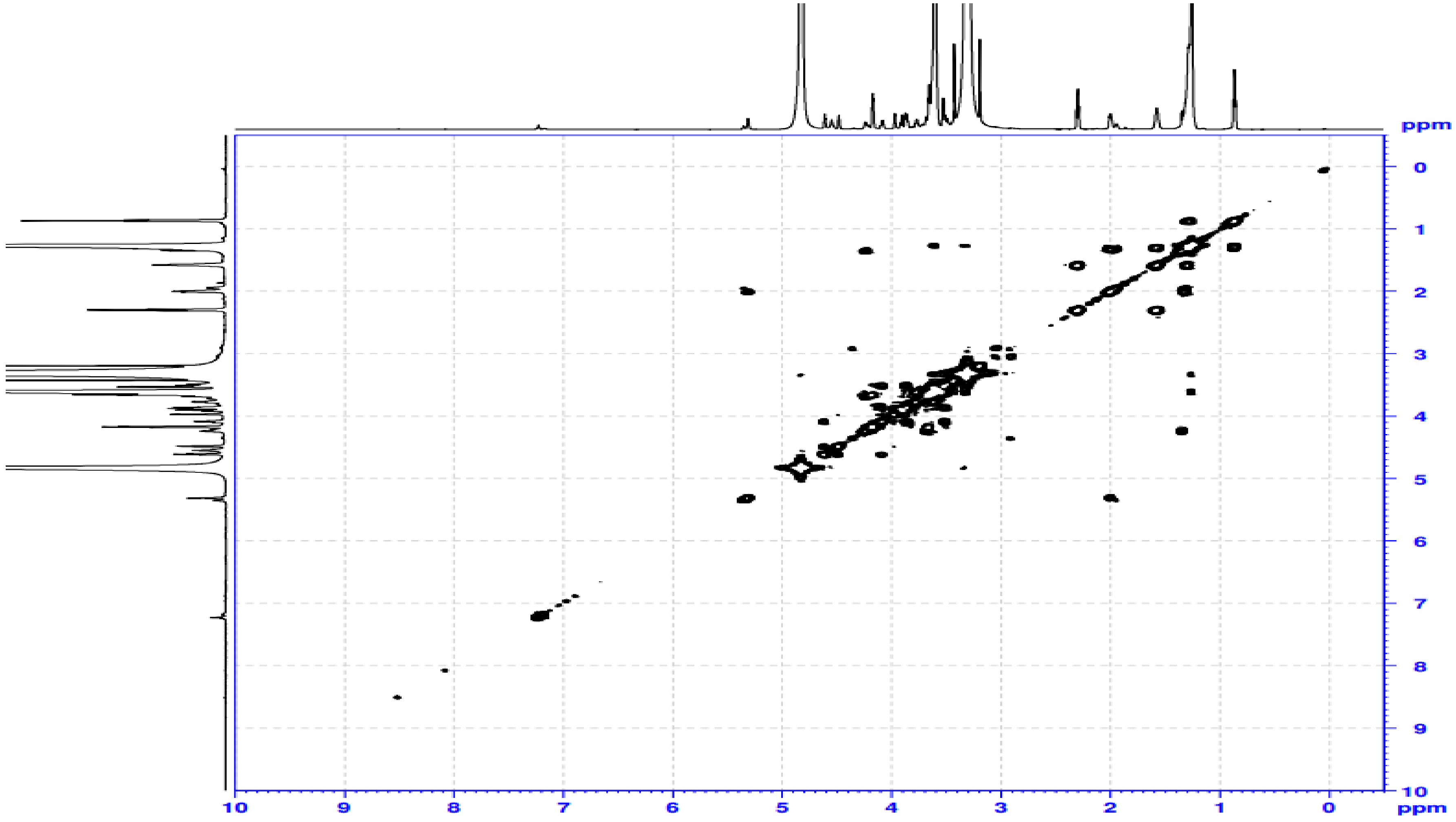

Figure S13 HR-FAB/MS spectrum of compound 2 in CD_3OD

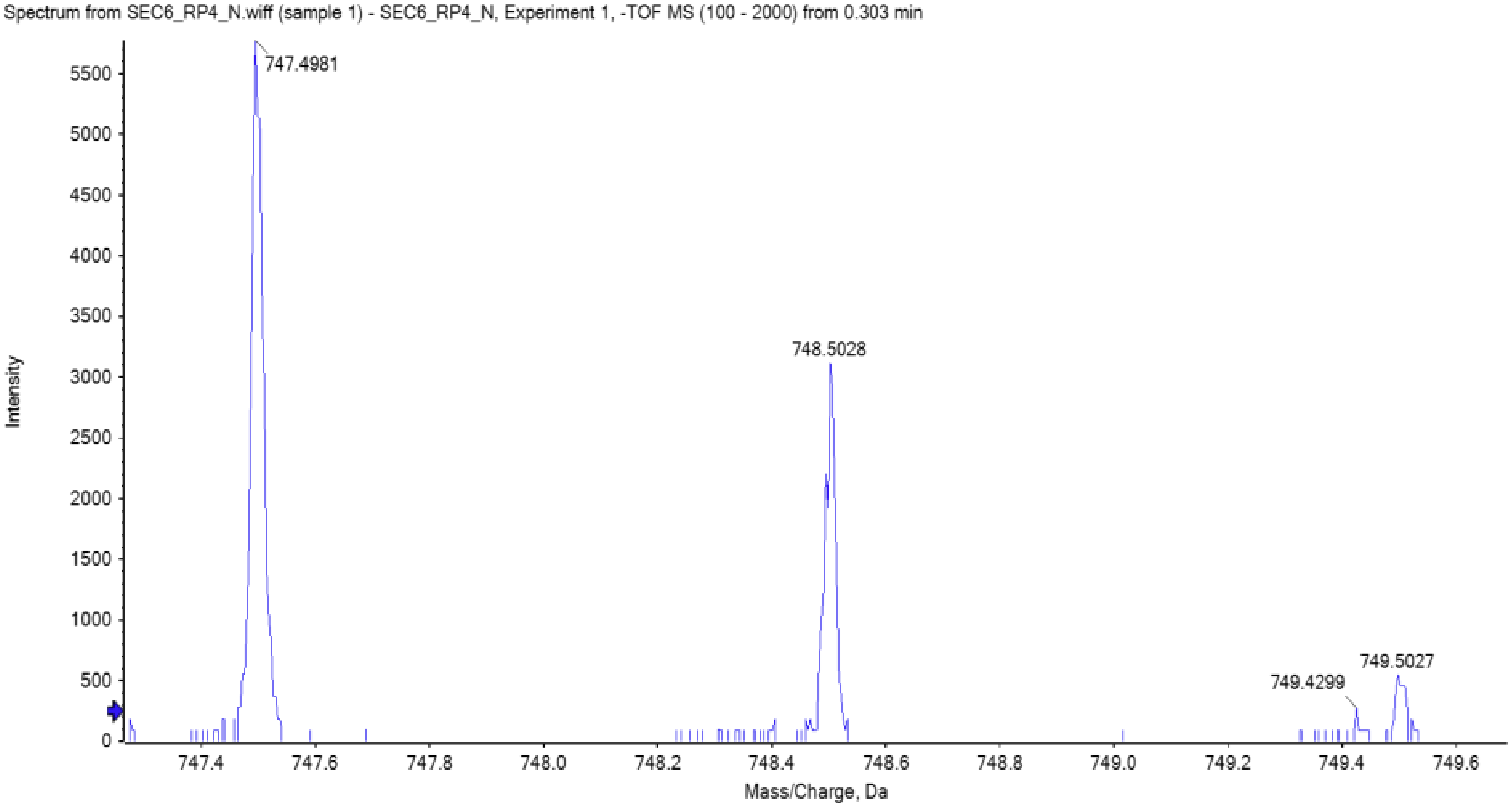

Supplement: Supplemental file 1 — Supplemental material. Download spectrum.01122-22-s0001.pdf, PDF file, 0.8 MB [file spectrum.01122-22-s0001.pdf]
